# In Vitro Antidiabetic, Antioxidant, and Prebiotic Activities of the Chemical Compounds Isolated from *Guizotia abyssinica*

**DOI:** 10.3390/antiox11122482

**Published:** 2022-12-16

**Authors:** Ahmed Elbermawi, Mohamed Samir Darwish, Ahmed A. Zaki, Noha A. Abou-Zeid, Mohamed A. Taher, Ebtihal Khojah, Somaiah A. Bokhari, Amal F. Soliman

**Affiliations:** 1 Department of Pharmacognosy, Faculty of Pharmacy, Mansoura University, Mansoura 35516, Egypt; 2 Dairy Department, Faculty of Agriculture, Mansoura University, Mansoura 35516, Egypt; 3 Veterinary Medicine Directorate, Mansoura 35516, Egypt; 4 Agricultural Chemistry Department, Faculty of Agriculture, Mansoura University, Mansoura 35516, Egypt; 5 Department of Food Science and Nutrition, College of Science, Taif University, P.O. Box 11099, Taif 21944, Saudi Arabia; 6 Pharmaceutical Care Department Maternity and Children Hospital, Mecca 24245, Saudi Arabia

**Keywords:** niger plant, antidiabetic activity, antioxidant activity, prebiotic activity

## Abstract

India and Ethiopia employ *Guizotia abyssinica* (niger plant) as a source of edible vegetable oil. Previous studies have documented the niger plant’s antioxidant properties and dietary benefits. Here, *G. abyssinica* extract was obtained and ten known bioactive components (**1**–**10**) were isolated. The antioxidant, antidiabetic, and prebiotic properties of whole extract and isolated components of niger and the plant’s ability to cooperate symbiotically with probiotic strains were examined. Compound **10**, myricetin-3-*O*-L-rhamnoside, had the highest antioxidant capacity measured in the 2,2-diphenylpicrylhydrazyl (DPPH, 4629.76 ± 6.02 µmol Trolox equivalent/g compound) and ferric-reducing antioxidant power (FRAP, 2667.62 ± 7.5 mol Trolox equivalent/g compound) assays. The lowest *α*-amylase and glycogen phosphorylase activities and glucose diffusion were obtained with whole *G. abyssinica* extracts, whereas compounds **8**–**10** had moderate inhibitory effects. *G. abyssinica* extract also induced the highest glucose absorption by yeast cells in the presence of 5 mM of glucose. Moreover, *Lactobacillus plantarum* and *L. rhamnosus* incubated with *β*-sitosterol 3-*O*-D-glucoside (compound **7**) showed the highest prebiotic activity score. The levels of L-(+)-lactic acid isomer in the probiotic strains were the highest in presence of the whole extract and decreased progressively in the presence of flavonoid glycosides (compounds **8**–**10**) and *β*-sitosterol 3-*O*-D-glucoside. The enzymatic profile of the probiotic strains was unaffected by the niger extract and compounds **7**–**10**. The findings revealed that the biological activities of *G. abyssinica* extract are mediated by the compounds **1**–**10**, and it may be considered as a promising plant for the treatment of diabetes mellitus**.**

## 1. Introduction

Diabetes mellitus is a very common disease affecting people in developing and developed countries. It was responsible for about 3.5% of deaths worldwide in 2012 [1]. Diabetes is classified into two types, i.e., type 1 (T1D) and type 2 (T2D) diabetes. It is considered that around 26.3% of Egyptian adults have T2D [2]. Diabetes is a metabolic disorder impairing protein, fat, carbohydrate, water, and electrolyte metabolism, mainly as a result of a disruption in insulin production and/or action [3]. T1D results from a deficiency in insulin secretion, which leads to hyperglycemia [4,5], whereas T2D is caused by a combination of insulin resistance and an inadequate insulin-secretion response [6].

Phytotherapy is increasingly being used in alternative medicine as a substitute for pharmaceuticals, particularly when scientific evidence of clinical significance supports its usage. Indeed, many natural substances used in conventional therapy have proven to be effective, occasionally have significantly fewer side effects, and are frequently inexpensive [7]. The pharmacological properties of phytochemicals for the treatment of numerous diseases, including diabetes, have been confirmed by numerous findings [8]. Many bioactive substances, including phenolic compounds, flavonoids, terpenoids, coumarins, and macromolecules, such as peptides, carbohydrates, and oils, have been linked to these therapeutic properties [8].

*Guizotia abyssinica* Cass. (niger plant) is an herbaceous green plant with vivid yellow flowers from the Asteraceae family. The niger plant is a source of edible seed oil and is cultivated in Ethiopia’s highlands and India; thus, it constitutes an important element of these countries’ economies [9]. Because *G. abyssinica* provides 50–60% of Ethiopia’s edible oil needs, research typically focuses on improving its crop biomass and oil yield. There are six species in the genus *Guizotia*, five of which are native to the highlands of Ethiopia, including Niger [10]. Tanzania, Bangladesh, the West Indies, Nepal, Zimbabwe, Malawi, Uganda, Zaire, and Sudan all have a limited supply of niger seeds [11].

*Guizotia. abyssinica* is a significant oil crop in Ethiopia because of its high amount of linoleic acid (55–80%). This unsaturated fatty acid has several medical benefits, including preventing heart diseases [12]. The ability of extracts from niger seed variants to substitute synthetic chemicals is likely due to their antioxidant capability [13]. Although *G. abyssinica* has been widely investigated for the quality of its oil seeds and its fatty acid content, which both positively affect human health, the phytochemical properties of *G. abyssinica*’s aerial parts have been little studied. Only nine flavonoids have been found in the aerial parts of *G. abyssinica* growing in Taiwan [14].

Metabolic enzymes specific to glycogenolysis and gluconeogenesis are used in several cutting-edge medical treatments of hepatic dysfunctions. Additionally, inhibitors of glycogen phosphorylase (EC 2.4.1.1) constitute a novel liver-targeted approach for the treatment of T2D. Many natural compounds have been reported to reduce the activity of glycogen phosphorylase [15]. In addition, *α*-amylase is an essential enzyme responsible for the digestion of starch, playing a key role in determining the amount of glucose released. Dietary polyphenols are recommended for controlling postprandial hyperglycemia because they slow down starch digestion by inhibiting *α*-amylase activity [16]. There have been reports of an antidiabetic effect from the ethanolic extract of *G. abyssinica* leaves. In streptozotocin-induced diabetic Albino Wistar rats, the *G. abyssinica* extract reduced the blood glucose levels and induced the dose-dependent inhibition of *α*-amylase [17]. 

Prebiotics are a group of chemicals known for more than 25 years that can alter the gut microbiota and benefit the host’s health. Prebiotics are now defined differently than they were then. According to the latest definition of prebiotics, polyphenols are a novel class of prebiotics, as they satisfy the requirements for being designated as prebiotic substrates [18].

There is no information available on the chemistry of the niger plant’s aerial parts. Therefore, the present study aimed to investigate the antioxidant activity of compounds from the niger plant’s aerial parts using the 2,2-diphenylpicrylhydrazyl (DPPH) and ferric-reducing antioxidant power (FRAP) assays. Moreover, the antidiabetic activity of the isolated compounds was evaluated by determining their inhibitory effect on the *α*-amylase and glycogen phosphorylase activities and their impact on glucose diffusion and uptake by yeast. The prebiotic activities of *G. abyssinica* extract and isolated compounds were also investigated by assessing their effects on parameters such as the growth rate, production of optically active forms of lactic acid, and enzymatic profile in probiotic strains.

## 2. Materials and Methods

### 2.1. Material

#### 2.1.1. Plant Material

Aerial parts of *G. abyssinica* Cass (Asteraceae) were collected in July 2015 from farmers’ fields in Oromia State, Ethiopia (7.55° N, 40.63° E). Staff at the Holeta Agricultural Research Center verified the identity of the plants. The aerial parts were air-dried in the shade at room temperature. A voucher specimen with the code Gu/003 was kept at the Pharmacognosy Department, Faculty of Pharmacy, Mansoura University.

#### 2.1.2. Chemicals

The following chemicals and reagents were purchased from Sigma-Aldrich Corporation (St. Louis, MO, USA): phosphorylated glycogen phosphorylase from rabbit muscle (mGPa), 5-chloro-N-[(1S,2R)-3-(dimethylamino)-2-hydroxy-3-oxo-1-(phenylmethyl)propyl]-1H-indole-2-carboxamide (CP-91149), (±)-6-hydroxy-2,5,7,8-tetramethylchromone-2-carboxylic acid (Trolox), 2,2-diphenyl-1-picrylhidrazyl radical (DPPH·), 2,4,6-tris(2-pyridyl)-s-triazine (TPTZ), ferric chloride, dimethylsulfoxide (DMSO), glycogen, glucose 1-phosphate, MgCl_2_, 4-(2-hydroxyethyl)-1-piperazineethanesulfonic acid (HEPES), 3,5-dinitrosalicylic acid (DNS), acarbose, and *α*-amylase. The reagent BIOMOL^®^ Green used for phosphate colorimetric measurement was bought from Enzo Life Sciences, Inc. (Postfach, Lausen, Switzerland). 

#### 2.1.3. Strains and Starter Culture 

All bacterial strains—*Lactobacillus plantarum MSD74*, *Lactobacillus rhamnosus* ML57, *Lactobacillus paracasei* MSD108, and *Escherichia coli* K12—were obtained from the microbiology laboratory’s culture collection (Dairy department, Faculty of Agriculture, Mansoura University, Egypt). The bacterial culture media, including nutrient agar, nutrient broth, de Man, Rogosa, and Sharpe (MRS) broth, and MRS agar, were purchased from Thermo Fisher Scientific (Cairo, Egypt). 

### 2.2. Extraction and Isolation

Seven hundred and twenty-five grams of powdered, air-dried aerial parts was extracted at room temperature by maceration with 90% MeOH. The solvent-free extract (83.3 g) was successively fractionated with petroleum ether, methylene chloride, ethyl acetate, and *n*-butanol. A silica gel column produced in petroleum ether (2 × 70 cm) was used for the chromatography of the petroleum ether fraction (5.25 g). A petroleum ether–ethyl acetate gradient was applied to the column to produce 5 fractions (1–5), ranging from 100:0 to 75:25 *v*/*v*. To obtain sub-fraction 50 comprising compound **1** and other impurities (11.4 mg), fraction 2 (244 mg) was exposed to a silica gel column (50 × 1.5 cm) using gradient elution with petroleum ether–methylene chloride (70:30 to 50:50 *v*/*v*), followed by further purification over Sephadex LH 20 to produce compound **1** (6.4 mg). Fraction 3 (43.5 mg) was subjected to chromatographic purification using Sephadex LH 20 to yield compound **2** (3.3 mg). By crystallization, fraction 4 (37.6 mg) was purified to produce compound **3**, *β*-sitosterol (5.6 mg).

Using a wet method, a silica gel column (2.5 × 75 cm) previously packed in *n*-hexane was used for the chromatography of the methylene chloride fraction (7.63 g). Gradient elution was used, commencing with n-hexane and progressing through EtOAc concentrations with 5% increments until 100% EtOAc was reached. Then, 10% EtOAc in *n*-hexane was used to elute the fractions (20–24). After heating with the vanillin–sulfuric acid spray reagent, the TLC plate showed a single brown spot at R*_f_* = 0.65 in a 20% EtOAc/*n*-hexane solvent solution. The collected fractions were evaporated to dryness, and compound **4**, a white, waxy material, was obtained (125 mg). By using 15% EtOAc in *n*-hexane, fractions (36–41) were eluted. After being heated with the vanillin–sulfuric acid spray reagent, the TLC plate showed a single yellow spot at R*_f_* = 0.43 in a 20% EtOAc/*n*-hexane solvent solution. Compound **5**, a yellow, amorphous material, was produced by drying the collected fractions by evaporation (30 mg). Fractions (52–71) were eluted by 20% EtOAc in *n*-hexane, and a TLC plate, heated with the vanillin–sulfuric acid spray reagent, showed various impurities, including a prominent violet spot at R*_f_* = 0.52 in a 50% EtOAc/*n*-hexane solvent solution. Using an isocratic mobile phase of EtOAc/*n*-hexane 18%, these fractions were pooled and re-chromatographed on normal-phase silica gel CC, producing compound **6** (250 mg). After heating with the vanillin–sulfuric acid spray reagent, fractions (111–119) were eluted using 50% EtOAc in n-hexane, and a TLC plate exhibited a significant violet spot with R*_f_* = 0.45 in a 10% MeOH/chloroform solvent solution. Compound **7**, a white, amorphous material, was produced by drying the collected fractions through evaporation (90 mg).

The petroleum ether-prepared silica gel column (450 cm) was used to chromatograph the ethyl acetate fraction (8.55 g). Four fractions (1–4) were obtained by running the column with a gradient of petroleum ether–ethyl acetate from 80:20 to 0:100 *v*/*v*. Fraction 1 was purified by crystallization to produce compound **8** (7.1 mg). After further purifying subfractions 25–27 on Sephadex LH 20 using 100% methanol as an eluent, compound **9** (11.2 mg) in its pure form was isolated from fraction 2 (273 mg) by re-chromatography utilizing the isocratic mobile phase of petroleum ether–ethyl acetate (50:50). Silica gel CC was applied to fraction 4; the elution gradient with methylene chloride–methanol was from 90:10 to 80:20 *v*/*v*; then, using methanol 100% as an eluent, further purification of the sub-fractions 51–57 (56 mg) using Sephadex LH 20 was performed to isolate compound **10** (14.6 mg).

### 2.3. General Procedures for Compounds’ Identification

On a Mattson 5000 FTIR, KBr-pellet IR spectra were captured (England). The coupling constants (J values) were stated in Hertz, and the ^1^H and ^13^C-NMR spectra were scanned on Bruker Ascend TM spectrometer (400 MHz) equipment using TMS as an internal standard (Hz). LC-MS-IT-TOF was used to determine HR-ESI-MS (Shimadzu, Tokyo, Japan). Survey scans were taken using the MS instrument employing an ESI source in both the positive and negative ionization modes with *m*/*z* 100–2000 for MS and *m*/*z* 50–1500 for MS/MS. The ionization parameters were as follows: the heat block temperature was 200 °C, the CDL temperature was 200 °C, and the probe voltage was 4.5 kV. The nebulizer gas flow was 1.5 L/min. Column chromatography was performed on Sephadex LH-20 (Amersham Pharmacia Biotech) and silica gel (60–200 microns; Merck, Germany). TLC (Merck or Machery-Nagel, Germany) was performed on precoated plates coated with silica gel 60 GF 254 and observed by UV light, vanillin–sulfuric acid reagent spraying, and heating at 110 °C for 5–10 min.

**Pentyl ferulate** (**1**); colorless powder, HRESIMS (Figure S1) *m*/*z* 301.1513 [M+Na]^+^ (calcd, 301.1416). ^1^H-NMR (CDCl_3_, 400 MHz, Figure S2): *δ* 7.54 (1H, *d*, *J* = 12.8 Hz, H7), 6.99 (2H, *m*, H5, 6), 6.95 (1H, *d*, *J* = 8.1 Hz, H2), 6.22 (1H, *d*, *J* = 12.8 Hz, H8), 4.10 (2H, t, *J* = 6.8 Hz, H1`), 3.88 (3H, *s*, OCH_3_), 1.63 (2H, *m*, H2`), 1.18 (6H, *m*, H3`,4`,5`), 0.78 (3H, *t*, *J* = 6.8 Hz, H6`). APT NMR (100 MHz, CDCl_3_, Figure S3) δ 167.4 (C9), 147.9 (C4), 146.8 (C3), 144.7 (C7), 127.1 (C1), 123.1 (C6), 115.7 (C8), 114.7 (C2), 109.3 (C5), 64.6 (C1`), 55.9 (OCH_3_/C3`), 31.9 (C4`), 28.7 (C2`), 25.8 (C3`), 22.7 (C5`), and 14.2 (C6`). The HSQC and HMBC spectra are presented in Figures S4 and S5, respectively.

**Sesamin** (**2**); colorless prisms. ^1^H-NMR (CDCl_3_, 400 MHz, Figure S6): *δ* 7.19 (2H, *s*, H2, 2`), 6.78 (2H, *br s*, H6, 6`), 6.72 (2H, *br s*, H5, 5`), 5.88 (2H, *s*, OCH_2_O), 4.64 (2H, *d*, *J* =3.48 Hz, H7, 7`), 4.16 (*m*, 2H, H9a, 9`a), 3.79 (*dd*, *J* = 9.0, 6.6 Hz, 2H, H9b,9`b), and 2.98 (2H, *m*, H8,8`). APT-NMR (CDCl_3_, 100 MHz, Figure S7): *δ* 147.5 (C3, C3`), 147.1 (C4, C4`), 135.0 (C1, C1`), 119.4 (C6, C6`), 108.2 (C5, C5`), 106.5 (C2, C2`), 101.1 (OCH_2_O), 85.8 (C7, C7`), 71.7 (C9, C9`), and 54.3 (C8, C8`) [19]. The HSQC and HMBC spectra are presented in Figures S8 and S9, respectively.

***β*-Sitosterol** (**3**); white powder eluted with petroleum ether–ethyl acetate (93:7). IR ʋ_max_ cm^−1^ (KBr): 3448, 2962, 1652, 1465, 1390, 1072, and 977 cm^−1^. ^1^H-NMR (CDCl_3_, 400 MHz, Figure S10): *δ* 0.6 (3H, *s*, H28), 0.73 (3H, *m*, H27), 0.75 (3H, *m*, H26), 0.77 (3H, *m*, H24), 0.85 (3H, *d*, *J* =6.4, H19), 0.93 (3H, *s*, H29), 3.46 (1H, *m*, H3), and 5.29 (1H, *m*, H5).

**1-Heneicosanol (4)**; white waxy substance. IR ʋ_max_ cm^−1^ (KBr, Figure S11): 3330, 2852–2920, 1540, 1072, and 975 cm^−1^. Its FAB-ESI-MS spectrum (Figure S12) showed a molecular ion peak at *m/z* 311 [M-H]^−^, corresponding to the molecular formula of C_21_H_44_O. ^1^H-NMR (CDCl_3_, 400 MHz, Figure S13): *δ* 3.57 (2H, *t*, H1), 1.50 (2H, *m*, H2), 1.18 (36H, *br.s*, H3–H20), and 0.81 (3H, *t*, H21). APT-NMR (CDCl_3_, 100 MHz, Figure S14): *δ* 63.1 (C1), 32.8 (C2), 31,9 (C19), 29.6 (C4–C18), 25.7 (C3), 22.7 (C20), and 14.1 (C21).

**Genkwanin (5)**; yellow amorphous powder. Its HRESIMS spectrum (Figure S15) showed a molecular ion peak at *m*/*z* 283.0631 [M-H]^−^ (calcd, 284.0606). ^1^H-NMR (MeOD, 400 MHz, Figure S16): *δ* 7.47 (*d*, *J* = 8.7 Hz, 2H, H2`,6`), 6.88 (*d*, *J* = 8.7, 2H, H3`,5`), 6.49 (*s,* 1H, H3), 6.47 (*d*, *J* = 2, 1H, H8), 6.28 (*d*, *J* = 2, 1H, H6), and 3.81 (*s*, 3H, OCH_3_). APT-NMR (100 MHz, MeOD, Figure S17): *δ* 182.6 (C4), 165.6 (C7), 165.1 (C2), 161.2 (C4`), 161.1 (C5), 157.8 (C9), 128.3 (C2`,6`),121.9 (C1`), 115.9 (C3`, 5`), 103.2 (C3, 10), 98.1 (C6), 92.6 (C8), and 55.6 (OCH_3_) [20,21]. The HMBC spectrum and its expansion are presented in Figures S18 and S19, respectively.

**(-) Epi-pinoresinol (6)**; yellow resinous substance. FAB-ESI-MS spectrum (Figure S20): *m/z* 357.0774 [M-H]^−^. ^1^H-NMR (CDCl_3_, 400 MHz, Figure S21): *δ* 6.87–6.69 (*m*, 6H, aromatic H, 2, 2′, 5, 5′, 6, and 6′), 5.60 (*br.s*, 2 OH, quenched by the addition of D_2_O), 4.79 (*d*, *J* = 5.2, 1H, H7`), 4.35 (*d*, *J* = 7.1, 1H, H7), 3.84 (*s*, 3H, OCH_3_), 3.82 (*s*, 3H, OCH_3_), 3,76 (*m*, 2H, H9, 9`), 3,25, and 2.84 (*m*, 2H, H8, 8′). APT-NMR (CDCl_3_, 100 MHz, Figure S22): *δ* 146.7 (C3`), 146.4 (C3), 145.3 (C4`), 144.5 (C4), 132.9 (C1`), 130.5 (C1), 119.2 (C6), 118.4 (C6`), 114.2 (C5, C5`), 108.5 (C2`), 108.3 (C2), 87.7 (C7`), 82.1 (C7), 70.9 (C9`), 69.7 (C9), 55.9 (2 OCH_3_), 54.4 (C8`), and 50.1 (C8) [22]. The HSQC and HMBC spectra are presented in Figures S23 and S24, respectively.

***β*-Sitosterol 3-*O*-*β*-D-glucoside (7)**; white amorphous powder. IR ʋ_max_ cm^−1^ (KBr): 3419, 2958, 2871, 1650, 1462, 1374, 1259, 1164, 1073, and 1024 cm^−1^. ^1^H-NMR (MeOD, 400 MHz, Figure S25): *δ* 5.27 (*d*, *J* = 4, 1H, H6), 4.32 (*d*, *J* = 7.7, 1H, H1′), 3.98–3.20 (H and OH of glucose H2′-H6′), 3.13 (*m*, 1H, H3), 2.33 (*m*, 1H, H4a), 2.18 (*m,* 1H, H4b), 1.95–1.77 (*m*, 4H, H7, 16a, 25), 1.58 (*m*, 2H, H2), 1.51 (*m*, 2H, H11), 1.48 (*m*, 2H, H12), 1.41 (*m*, 1H, H20), 1.36 (*m*, 3H, H8, 28), 1.28–1.20 (*m*, 6H, H16b, 17, 22, 23), 1.17 (*s*, 3H, H29), 1.08 (*m*, 2H, H1), 1.04 (*m*, 2H, H15), 0.96 (*m*, 2H, H14, 24), 0.93 (*s*, 3H, H19), 0.85 (*d*, *J* = 6.3, 3H, H21), 0.74 (*m*, 1H, H9), 0.76 (*d*, *J* = 8.6, 3H, H27), 0.72 (*d*, *J* = 8.6, 3H, H26), and 0.60 (*s*, 3H, H18). APT-NMR (100 MHz, MeOD, Figure S26): *δ* 140.2 (C5), 121.9 (C6), 101.0 (C1`), 78.9 (C3), 76.4 (C3`), 75.9 (C5`), 73.5 (C2`), 70.1 (C4`), 61.7 (C6`), 56.6 (C14), 55.9 (C17), 50.1 (C9), 45.7 (C24), 42.2 (C13), 39.6 (C4), 38.5 (C12), 37.1 (C1), 36.6 (C10), 36.0 (C20), 33.8 (C22), 31.8 (C7, 8), 29.4 (C2), 29.03 (C25), 28.1 (C16), 25.9 (C23), 24.1 (C15), 22.9 (C28), 20.9 (C11), 19.5 (C26), 19.1 (C19), 19.07 (C27), 18.7 (C27), 18.5 (C21), and 11.6 (C18, 29) [23]. 

**6`-*O*-(4``-hydroxy-*trans*-cinnamoyl)-kaempferol-3-*O*-*β*-D-glucopyranoside** (**8**); yellowish gum; ^1^H NMR (400 MHz, MeOD, Figure S27): *δ* 7.89 (*m*, 2H, H2`,6`), 7.30 (*d*, *J* = 15.9 Hz, 1H, H7```), 7.21 (*m*, 2H, H2```, 6```), 6.71 (*m*, 4H, H3`, 5`, 3```, 5```), 6.21 (*d*, *J* = 2.1 Hz, 1H, H6), 6.03 (*d*, *J* = 2.1 Hz, 1H, H8), 5.97 (*d*, *J* = 15.9 Hz, 1H, H8```), 5.15 (m, 1H, H1``), 4.22 (*dd*, *J* = 11.9, 2.3 Hz, 1H, H6``a), 4.09 (*dd*, *J* = 11.8, 6.6 Hz, 1H, H6``b), 3.37 (*m*, 3H, H3``, 5``, 2``), and 3.21 (*m*, 1H, H4``). APT-NMR (100 MHz, MeOD, Figure S28): *δ* 178.0 (C4), 167.4 (C9```), 164.5 (C7), 161.6 (C5), 160.1 (C4`), 159.8 (C4```), 158.0 (C2), 157.0 (C9), 145.2 (C7```), 133.8 (C3), 130.8 (C2`, 6`), 129.8 (C2```, 6```), 125.7 (C1```), 121.3 (C1`), 115.4 (C3`, 5`), 114.7 (C3```, 5```), 113.3 (C8```), 104.2 (C10), 102.5 (C1``), 98.6 (C6), 93.4 (C8), 76.6 (C5``), 74.4 (C3``), 74.3 (C2``), 70.3 (C4``), and 62.9 (C6``) [24]. The HSQC and HMBC spectra are presented in Figures S29 and S30, respectively.

**Tamarixetin-3-*O*-*β*-D-glucoside** (**9**); yellow amorphous powder. ^1^H NMR (400 MHz, MeOD, Figure S31): *δ* 7.83 (*d*, *J* = 1.9 Hz, 1H, H2`), 7.49 (*dd*, *J* = 8.4, 2.0 Hz, 1H, H6`), 6.81 (*d*, *J* = 8.2 Hz, 1H, H5`), 6.30 (*d*, *J* = 1.9 Hz, 1H, H8), 6.10 (*d*, *J* = 2.0 Hz, 1H, H6), 5.29 (*d*, *J* = 6.6 Hz, 1H, H1``), 3.85 (*s*, 3H, OCH_3_/4`), 3.62 (m, 1H, H5``), 3.41 (m, 3H, H3``, 6``), and 3.21 (*m*, 2H, H2``, 4``). APT-NMR (MeOD, 100 MHz, Figure S32): *δ* 177.8 (C4), 164.4 (C7), 161.5 (C5), 157.1 (C9), 156.9 (C2), 149.3 (C3`), 146.8 (C4`), 133.8 (C3), 122.2 (C6`), 121.5 (C1`), 114.5 (C5`), 112.8 (C2`), 104.2 (C10), 102.1 (C1``), 98.3 (C6), 93.2 (C8), 76.9 (C3``), 76.4 (C5``), 74.3 (C2``), 69.9 (C4``), 60.9 (C6``), and 55.2 (OCH_3_/4`) [25]. The HSQC and HMBC spectra are presented in Figures S33 and S34, respectively.

**Myricetin-3-*O*-*α*-L-rhamnosid** (**10**); greenish-yellow powder. ^1^H NMR (400 MHz, MeOD, Figure S35): *δ* 6.85 (*s*, 2H, H2`, 6`), 6.26 (*d*, *J* = 2.1 Hz, 1H, H8), 6.10 (*d*, *J* = 2.1 Hz, 1H, H6), 5.21 (*d*, *J* = 1.7 Hz, 1H, H1``), 4.12 (*dd*, *J* = 3.3, 1.7, Hz, 1H, H2``), 3.69 (*dd*, *J* = 9.5, 3.4 Hz, 1H, H3``), 3.42 (*m*, 1H, H5``), 3.25 (*d*, *J* = 9.6 Hz, 2H, H2``, 4``), and 0.86 (*d*, *J* = 6.8 Hz, 3H, H6``). APT-NMR (MeOD, 100 MHz, Figure S36): *δ* 178.3 (C4), 164.7 (C7), 161.8 (C5), 158.0 (C2), 157.1 (C9), 145.5 (C3`), 136.5 (C4`), 134.9 (C3), 120.5 (C1`), 108.1 (C2`, 6`), 104.4 (C10), 102.2 (C1``), 98.5 (C6), 94.3 (C8), 71.9 (C4``), 70.7 (C3``), 70.6 (C5``), 70.5 (C2``), and 16.3 (C6``) [26].

### 2.4. In Vitro Assay of G. abyssinica Extract and Isolated Bioactive Compounds to Be Used as Ingredients of Synbiotic Preparations 

#### 2.4.1. Prebiotic Activity Score (A_preb_) of Selected Probiotic Strains 

The effects of compounds (**1**–**10**) and *G. abyssinica* extract on the ***A_preb_*** of probiotic strains were investigated. The overnight monocultures of selected probiotic strains and *Escherichia coli* K12 (K12) were used to inoculate MRS and nutrient broth (NB) (2% *v/v*), respectively, wherein 2% of the glucose was replaced with each compound individually. The inoculated media were incubated at 37 °C for 24 h. Subsequently, serial dilutions of the resultant bacterial growth were prepared, and after 24 h of incubation at 37 °C, the population of probiotic bacteria in MRS agar was counted using the pour plate method [27]. MRS broths without glucose or with 2% of glucose and inulin were used as the negative control, positive control, and prebiotic standard, respectively. A_preb_ was calculated as previously reported by Dawood, et al [28].

(1) $$\mathrm{Apreb}=\frac{\left(\mathrm{Log}\;\left(\mathrm{Lb}\right)24-\;\mathrm{Log}\;\left(\mathrm{Lb}\right)\;0\right)\;\mathrm{PC}}{\left(\mathrm{Log}\left(\mathrm{Lb}\right)\;24-\;\mathrm{Log}\;\left(\mathrm{Lb}\right)0\right)\;\mathrm{Glucose}\;}-\frac{\left(\mathrm{Log}\;\left(K12\right)24-\;\mathrm{Log}\;\left(K12\right)\;0\right)\;\mathrm{PC}}{\left(\mathrm{Log}\;\left(K12\right)24-\;\mathrm{Log}\;\left(K12\right)\;0\right)\;\mathrm{Glucose}\;}$$

The prebiotic activity score is represented by A_preb_, and Log Lb is the log CFU/mL of selected *Lactobacillus* spp. strains after 24 h (Lb24) and 0 h (Lb 0) of incubation at 37 °C. Log K12 represents the log of CFU/mL of K12 after 24 h ((K12) 24) and 0 h ((K12) 0) of culture on glucose and the isolated bioactive substances (PC). Based on the previous equation, the compounds or extract with high A_preb_ supported the tested probiotic strains’ growth when they allowed cell counts (CFU/mL) comparable to that found when grown on glucose.

#### 2.4.2. Assessment of Lactic Acid’s Optically Active Forms

Using a kit of D-/L-lactic acid (Megazyme, Ireland), calculations were performed to determine the ratio of each optically active type of lactic acid produced in monoculture by the studied strains. The manufacturer’s manual was followed for the testing procedure. Briefly, *G. abyssinica* extract and isolated compounds were added separately at 2% (*w*/*v*) to MRS broth medium in place of dextrose. The modified MRS broth was inoculated with an overnight monoculture of the selected strains (2% *v*/*v*), followed by incubation at 37 °C for 24 h. Then, the supernatants (50 µL) obtained from monocultures by centrifugation at 5000× *g* for 15 min were centrifuged. As a prebiotic standard and a positive control, MRS broth medium containing inulin or glucose was utilized, respectively. A buffer (250 µL) was added after each supernatant (50 µL) had been diluted with 750 µL of deionized water, 50 µL of NAD^+^ solution, and D-GPT (10 µL), followed by incubation at 37 °C for 3 min. The absorbance of the mixture was measured (Abs1) at 340 nm. Subsequently, 10 µL of D-LDH was transferred to the mixing tube. Samples were incubated at 37 °C for a further 5 min; thereafter, the Abs2 was measured at 340 nm; then, the L-LDH (10 µL) was added. After 10 min of incubation at 37 °C, the Abs3 of mixture samples was determined. Equation (2) was used to compute the concentrations of D- and L-lactic acid):

C = 0.3204 × ∆Abs D + 0.3232 × ∆Abs L(2)

C” indicates how much lactic acid there was in total. “0.3204 × ∆ODD” denotes the amount of the D-lactic acid, “0.3232 × ∆ODL” denotes the amount of the L (+) isomer, and ∆Abs D = Abs2 −Abs1 and ∆Abs L = Abs3−Abs2.

#### 2.4.3. The Effect of Isolated Bioactive Compounds or Extract on the Enzymatic Profile of Selected Strains

The enzymatic profiles of probiotic strains were determined as previously described by Śliżewska and Chlebicz-Wójcik [27]. The effects of isolated compounds (1–10) and extract on the enzymatic profile of selected probiotic strain were detected using a bioMérieux API ZYM test kit (Marcy l’Etoile, France). A 48-h incubation period at 37 °C was carried out after an overnight culture of the chosen strains was streaked onto MRS agar, with each isolated component (**1**–**10**) or extract individually at a concentration of 2% (*w*/*v*). To achieve a final density between 5 and 6 on McFarland’s scale, the monocultures were diluted in sterile saline solution (0.85%). All microtubes of the API^®^ ZYM test strip were individually filled with each suspension of the chosen strains, which were then incubated for 4 h at 37 °C. To each microtube, ZYM A and ZYM B reagents were added, and the test strips were then exposed to light. Positive results were obtained according to the changes in color in the microtubes. The dextrose and inulin were used as the positive control and standard prebiotic, respectively. 

### 2.5. In Vitro Evaluation of the Antioxidant Activity

The spectrophotometric DPPH and FRAP assays were performed as described by Yu*,* et al [29] to assess the antioxidant activity of the sample extracts. The absorbance at 520 nm was measured after incubating 190 µL of DPPH working solution (8.87 mM) in methanol and 10 µL of the sample dilutions (methanol was used as the blank) for 15 min at room temperature.

Acetate buffer (300 mM, pH 3.6), TPTZ (10 mM dissolved in hydrochloric acid), and FeCl_3_ (20 mM in water) were combined at a volume ratio of 10:1:1 to obtain the FRAP working solution. The mixture was kept at 37 °C for 10 min. Then, 180 µL of FRAP working solution was incubated with the sample dilutions (20 µL) at 37 °C for 30 min, and the absorbance was measured at 593 nm. The antioxidant activity assays were scaled down to the micron level using 96-well microplates and microplate readers (FluoStar Omega) with Trolox as the reference material. The unit of measurement for all data was micromoles of Trolox equivalent per gram of extract or compound (µmol Trolox equivalent (TE)/g extract or compound). 

### 2.6. Antidiabetic Properties of G. abyssinica Extract and Isolated Compounds

#### 2.6.1. In Vitro Glycogen Phosphorylase Inhibition Assay

The activity of glycogen phosphorylase (GPa) was determined using a previously validated and optimized method [30]. Specifically, 10 µL of the extract or isolated compounds (2 mg/mL in DMSO) was added to 50 µL of rabbit muscle GPa (0.38 UmL^−1^) and incubated at 37 °C for 15 min. Then, 45 µL of HEPES solution (50 mM; pH 7.2), containing 0.25 mg/mL glycogen, 0.25 mM glucose 1-phosphate, 2.5 mM MgCl_2_, and 100 mM KCl were added to the enzymatic reaction and further incubated at 37 °C for 15 min. The mixture was then incubated with 130 µL BIMOL Green, a reagent for colorimetric phosphate quantification, and the absorbance was measured spectrophotometrically at 620 nm. The inhibition of mGPa activity was determined as follows:

mGPa inhibition (%) = (B − A)/(C − A) × 100(3)

where A is the absorbance measured without enzyme (blank), B is the absorbance measured with the sample (i.e., enzyme and tested compounds), and C is the control absorbance (measured with the enzyme and without tested compounds). CP-91149 (3.13 M) was utilized as a positive control.

#### 2.6.2. Determination of the Inhibitory Effect of *G. abyssinica* Extract and Isolated Compounds on *α*-Amylase Activity

Elbermawi, et al [31] previously reported a method to measure the *α*-amylase inhibition rates. Briefly, 200 µL of extract or isolated chemicals (2 mg/mL) were incubated in 200 µL of 20 mM sodium phosphate buffer (pH 7.0, with 6 mM NaCl) that contained 10 U/L of *α*-amylase for 45 min at 37 °C. Then, 400 µL of a 0.5% potato starch solution was added to each sample, and the mixture was incubated for 10 min at 37 °C in a water bath that was shaken at 100 rpm. The enzymatic reaction was stopped by adding dinitrosalicylic acid color reagent. The test tubes were left in the water bath at 100 °C for 10 min and then cooled to room temperature. Lastly, 3 mL of deionized water was used to dilute the samples. Spectrophotometric measurements of the absorbance of the diluted reaction solutions were performed at 540 nm (Spectro UV–VIS Auto, UV2602, Labomed, Los Angeles, CA, USA). We compared and expressed the proportions of *α*-amylase inhibition in the control and test samples. Acarbose was used as positive control (5 g/mL).

#### 2.6.3. Determination of the Effects of Isolated Compounds and Extract on Glucose Diffusion

The rate of glucose diffusion was determined as reported by Hu, et al [32] with slight modifications. Briefly, the model used dialysis bags with molecular weight cutoffs of 8000–14,000 and 500–1000 to assess the effect of *G. abyssinica* extract and isolated compounds, respectively, on the rate of glucose diffusion. Ten milliliters of glucose solution (100 mM) and individual isolated compounds and extract (2 mg/mL) were transferred to dialysis bags. Dialysis was conducted against 100 mL of distilled water at 37 °C and pH 7.0. The concentration of glucose in 2 mL of the dialysate was determined after 20, 40, and 60 min as previously described by Miller [33]. 

#### 2.6.4. Effect of *G. abyssinica* Extract and Isolated Compounds on Glucose Uptake by Yeast Cells

Glucose uptake by commercial baker’s yeast was determined using the method described by Paul and Majumdar [34]. Briefly, commercial baker’s yeast was suspended in deionized water and incubated overnight at 25 °C. The yeast cell suspension was centrifuged at 5000× *g* for 10 min at 7 °C. The yeast cell pellet was resuspended in distilled water. The whole process was repeated until a clear supernatant was obtained. Then, 10 mL of yeast cell suspension was diluted with 90 mL of distilled water. Four hundred microliters of isolated *G. abyssinica* compounds or extract (2 mg/mL) were mixed with 1 mL of different concentrations of glucose (5, 10, and 25 mM). The mixture was inoculated with 100 µL of yeast suspension and incubated at 37 °C for 1 h. Then, the mixture was centrifuged at 4000× *g* for 7 min. The absorbance of the supernatant was measured at 520 nm using a spectrophotometer (Spectro UV–VIS Auto, UV2602, Labomed, USA). The glucose uptake by yeast cells was expressed as a percentage and calculated using the following Equation:

(4) $$\mathrm{Percentage}\;\mathrm{of}\;\mathrm{glucose}\;\mathrm{uptake}=\frac{\left(\mathrm{Absorbance}\;\mathrm{of}\;\mathrm{control}\;-\;\mathrm{Absorbance}\;\mathrm{of}\;\mathrm{sample}\right)}{\mathrm{Absorbance}\;\mathrm{of}\;\mathrm{control}\;}\times 100$$

## 3. Results

### 3.1. Identification of Isolated Compounds

**Figure 1:**
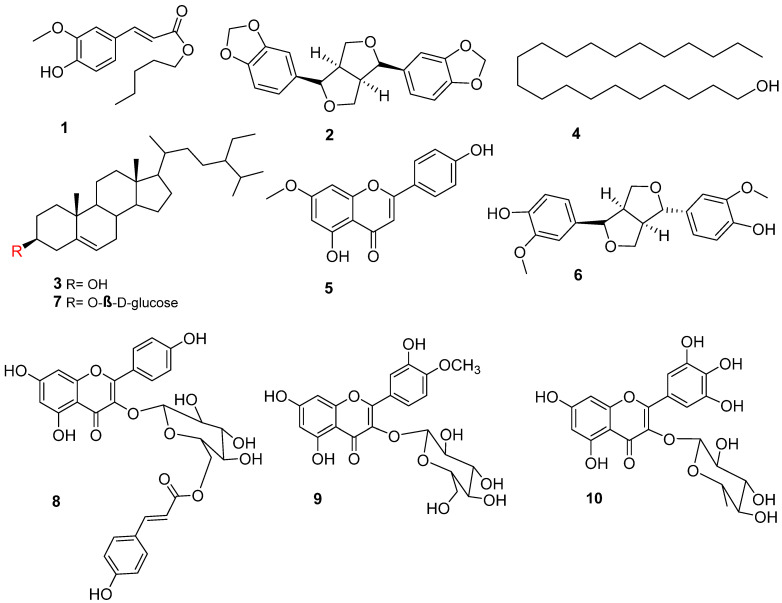
Structures of isolated compounds.

The phytochemical investigation of aerial parts of *G. abyssinica* led to the isolation of nine known compounds, namely sesamin (**2**), *β*-sitosterol (**3**), 1-heneicosanol **(4),** genkwanin (**5**), epipinoresinol (**6**), *β*-sitosterol 3-*O*-*β*-D-glucoside (**7**), 6`-*O*-(4``-hydroxy-*trans*-cinnamoyl)-kaempferol-3- *O*-*β*-D-glucopyranoside (**8**), tamarixetin-3-*O*-*β*-D-glucoside (**9**), and myricetin-3-*O*-*α*-L-rhamnosid (**10**), together with one new compound, pentyl ferulate (**1**) (Figure 1). Their structures were identified via the extensive analysis of both NMR and MS spectra. 

### 3.2. Evaluation of the Prebiotic Potential of G. abyssinica Extract and Isolated Compounds 

#### 3.2.1. Prebiotic Activity Scores (A_preb_) of *G. abyssinica* Extract and Isolated Compounds

**Figure 2:**
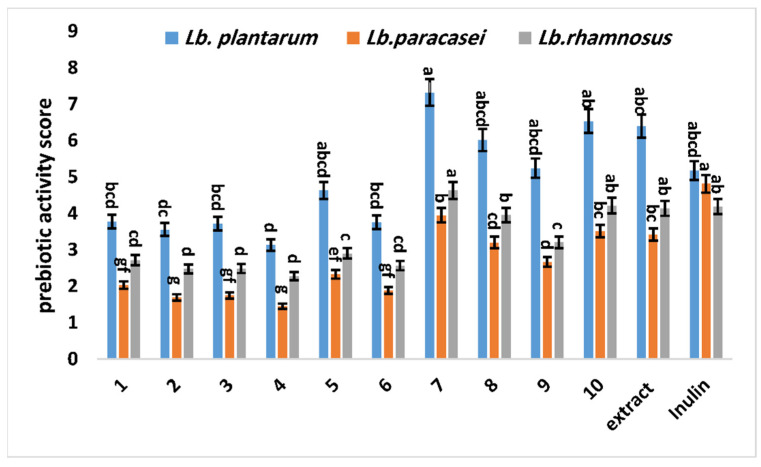
Activity scores (A_pre*b*_) of *Lactobacillus* spp. strains paired with different isolated compounds (**1**–**10**) and methanol extract. Lowercase letters show a significant impact on the prebiotic activity index (*p* ˂ 0.05). The standard deviations of the means of the treatments are shown by vertical bars.

In this investigation, *Lactobacillus* spp. were used as probiotic strains, and *E. coli* K12 was chosen as the enteric bacterium of A_preb_. Based on Equation (1), the values of A_preb_ displayed in Figure 2 were obtained from the growth of probiotic strains. All of the values of A_preb_ were positive, indicating that the growth rate of the selected probiotic strain with the isolated compounds or *G. abyssinica* extract was greater than the growth rate of *E. coli* K12 paired with the isolated compounds. The highest A_preb_ values were detected for *Lb. plantarum* and *Lb. rhamnosus* incubated with compound **7**, and the A_preb_ values of all isolated compounds and extracts paired with *Lb*. *paracasei* were lower than those of inulin (standard prebiotic). In contrast, low A_preb_ values for all tested strains were observed for compounds **2**–**4**. In this study, the A_preb_ values obtained with *Guizotia* extract were significantly (*p <* 0.05) higher than those obtained for isolated compounds (**1**–**6** and **9**) paired with *Lb paracasei* and *Lb. rhamnosus*. However, the values of A_preb_ related to *Lb. plantarum* indicate that the effect of *Guizotia* extract was significantly higher than that of compound **4**. Thus, a synergistic influence between the isolated compounds toward *Lactobacillus* spp. strains was likely. However, no significant differences between the A_preb_ values of the isolated compounds and extract were found (Figure 2).

#### 3.2.2. The Optical Type of Lactic Acid Synthesized by Selected *Lactobacillus* Strains 

**Figure 3:**
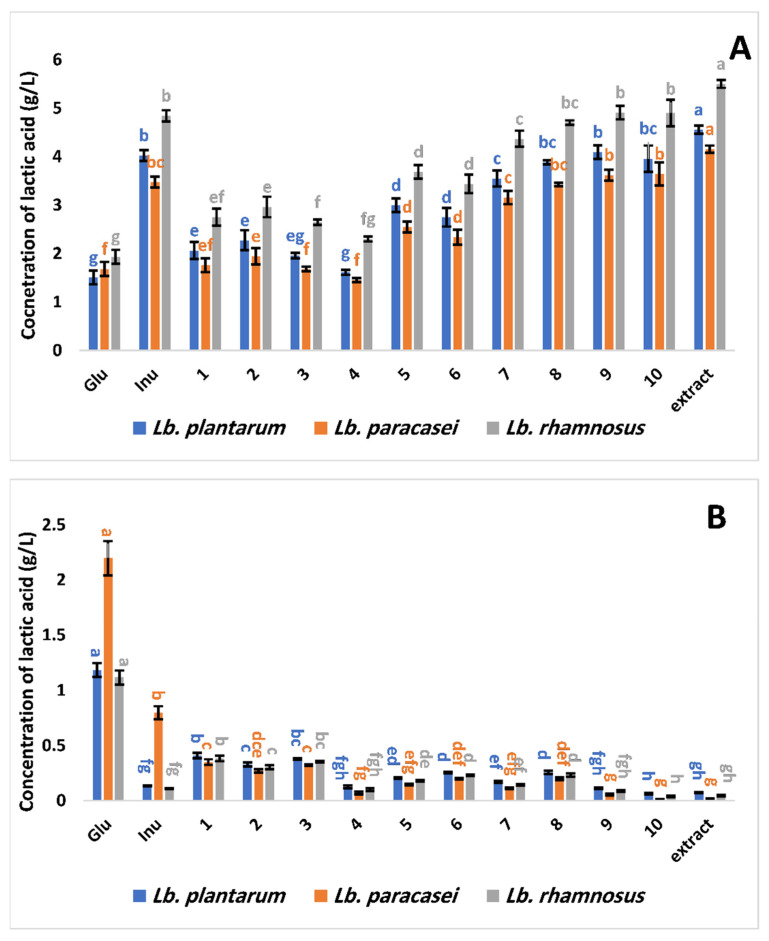
Effect of *G. abyssinica* extract and isolated bioactive compounds on probiotic bacteria’s ability to produce the optically active forms of lactic acid L (+) (**A**) and D (+) (**B**). Lowercase letters show a significant impact on the prebiotic activity index (*p* ˂ 0.05). The standard deviation between the means of the treatments is shown by vertical bars.

When the *Lactobacillus* spp. strains were cultivated in the presence of all of the compounds that were examined, and when glucose (a positive control) and inulin (a prebiotic standard) were utilized as the carbon source, both isomers of lactic acid, L (+) and D (−), were observed (Figure 3). The tested probiotic strains produced considerably more L (+) lactic acid when grown in the presence of isolated compounds (**2** and **5**–**10**) and extract than when glucose was the carbon source (Figure 3A). Moreover, when these all-tested substances or extract were utilized in comparison with glucose, a significant statistical decrease in the concentration of the D (−) isomer of this organic acid was found. (Figure 3B). The highest amount of the L (+) isomer of lactic acid was recorded for all selected probiotic strains in the presence of methanol extract, followed by, in descending order, flavonoidal glycosides (**8**–**10**) and **7** (Figure 3A). The presence of the glucose moiety at C3 increased the concentration of the L (+) isomer of lactic acid synthesized by the tested strains; with compound **7**, 3.16 to 4.37 g/L was produced, whereas, with compound **3**, the concentration ranged from 1.45 to 2.65 g/L (Figure 3A). Furthermore, no vital statistical differences in the concentrations of L (+) isomer of lactic acid were detected when the flavonoidal glycosides were used as the carbon source for the tested strains compared with inulin (prebiotic standard) (Figure 3A). The lowest concentration of the D (−) isomer of lactic acid was observed for *Lb. paracasei* in the presence of compound **10** (Figure 3B). 

#### 3.2.3. The Effects of Isolated Compounds and Methanol Extract on the Enzymatic Profiles of Probiotic Strains

**Figure 4:**
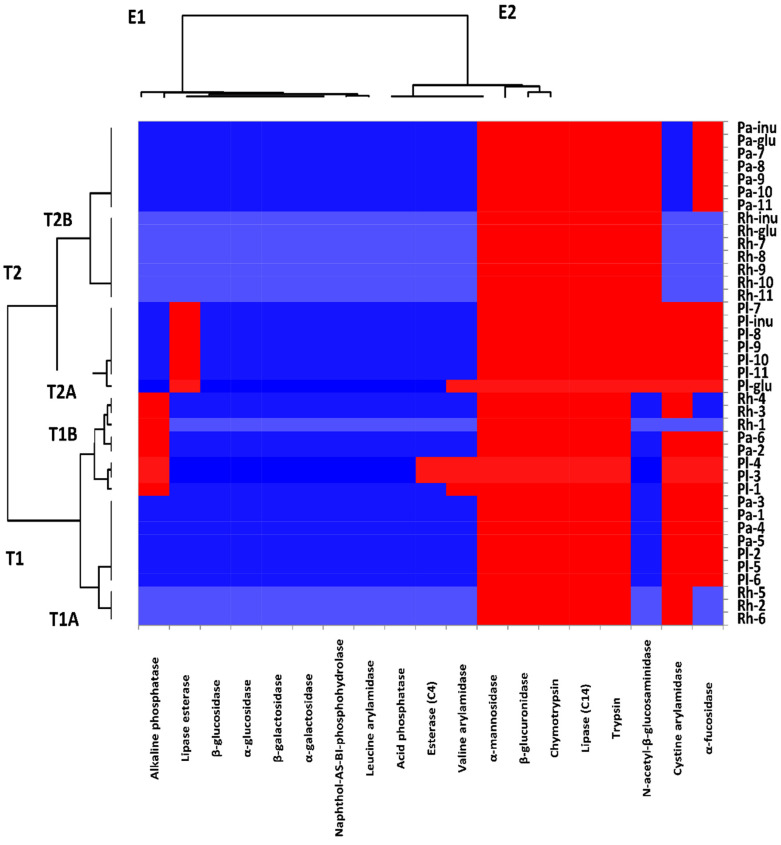
Heat map presenting the enzymatic profiles of the selected probiotic strains in relation to isolated phenolic compounds (**1**–**10**) or methanol extract. Glu and inu indicate glucose (positive control) and inulin (prebiotic standard), respectively. Rh, Pa, and Pl indicate *Lb. rhamnosus*, *Lb. paracasei*, and *Lb. plantarum*. Red and blue, respectively, indicate a lack of enzyme activity or a positive reaction of the enzymatic profile.

Heat maps were used to simultaneously analyze the sample clustering and features in a synthetic way (Figure 4). Hierarchical clustering analysis quantified the similarity levels between the enzymatic profile of probiotic strains and the same strains with the isolated phenolic compounds or extract as a carbon source by considering the distances between the possible pairs of carbon sources and enzymatic profiles. The hierarchical clustering classified treatments into clusters T1 and T2, corresponding to the type of carbon source. Cluster T1 included two subgroups: T1A indicated compounds **2**, **5**, and **6,** used as a carbon source for both *Lb. rhamnosus* and *Lb. plantarum*, and compounds **1** and **3**–**5,** used as a carbon source for *Lb. paracasei*. T1B indicated *Lb. rhamnosus* or *Lb. plantarum* paired with compounds **1**, **3**, and **4**, and *Lb. paracasei* associated with compounds **2** and **6**. Cluster T2 involved two subgroups: (T2A) *Lb. plantarum* paired with glucose (positive control), inulin (prebiotic standard), compounds **7**–**10,** or methanol extract. However, T2B indicated the same carbon source as T2A associated with *Lb. rhamnosus* and *Lb. paracasei* (Figure 4). The enzymatic profiles of these *Lactobacillus* spp. strains were grouped into two essential groups: E1 and E2 (Figure 4). 

It was noticed that each probiotic strain presented naphthol-AS-BI-phosphohydrolase, acid phosphatase, *β*-galactosidase, *α*-galactosidase, *β*-glucosidase, leucine arylamidase, and *α*-glucosidase activities independent of the compounds used as carbon sources for bacterial growth. However, the trypsin, lipase, chymotrypsin, *β*-glucuronidase, *β*-mannosidase, and *β*-fucosidase activities were absent in the studied strains that grew in the presence of glucose or isolated phenolic chemicals (except for *Lb. rhamnosus*). Furthermore, variations in the cystine arylamidase and alkaline phosphatase activities were found in the enzymatic profiles of the examined strains in relation to the isolated compounds used as carbon sources during growth. When pentyl ferulate, *ß*-sitosterol, and 1-heneicosanol were employed as carbon sources during cultivation, alkaline phosphatase activity was not observed in *Lb. rhamnosus* and *Lb. plantarum* (Figure 4). Alkaline phosphate was not present in the enzymatic profile of *Lb. paracasei* when sesamin and epipinoresinol were utilized as the carbon sources. However, when inulin (a prebiotic standard), extract, flavonoidal glycosides (**8**–**10**), and **7** were utilized as the carbon source, cystine arylamidase activity was found for *Lb. rhamnosus* and *Lb. paracasei*. 

### 3.3. Biological Activity of G. abyssinica Methanol Extract and Isolated Compounds

#### 3.3.1. Antioxidant Activity of *G. abyssinica* Extract and Isolated Compounds

Two common in vitro antioxidant assays (DPPH and FRAP) were used to assess the antioxidant activity of *G. abyssinica* extract and isolated compounds. The DPPH antiradical ability and FRAP values were expressed in µmol TE/g dried extract. Most isolated *G. abyssinica* compounds exhibited respectable antioxidant activity (Table 1). Compound **10** had the highest antioxidant capacity in the DPPH (4629.76 ± 6.02 µmol TE/g extract or compound) and FRAP (2667.62 ± 7.5 µmol TE/g extract or compound) assays. The second-most-effective antioxidant was compound **6**, whereas compound **4**, a long-chain primary fatty alcohol, exhibited the lowest antioxidant capacity in both assays.

#### 3.3.2. Antidiabetic Activity of *G. abyssinica* Extract and the Isolated Compounds

##### Inhibitory Effect on *α*-Amylase Activity

**Figure 5:**
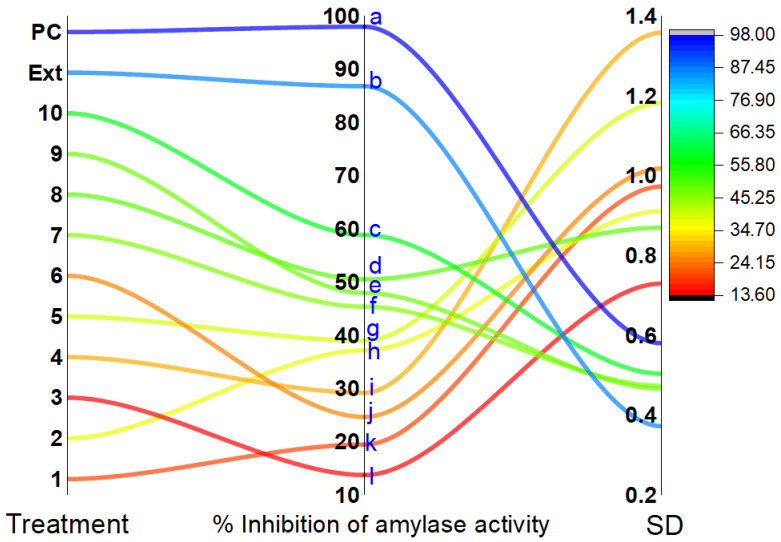
Effect of *G*. *abyssinica* extract (ext) and isolated bioactive compounds on *α*-amylase activity. Lower-case letters show a significant impact (*p* ˂ 0.05). Acarbose was used as a positive control (PC).

The *G. abyssinica* extract significantly decreased the *α*-amylase activity by 86.8 ± 0.37% (*p* < 0.05), and this inhibition was higher than that induced by the isolated compounds (Figure 5). Flavonoid glycosides (compounds **8**–**10**) induced 47.94–58.86% inhibition of *α*-amylase, whereas compound **5**—a flavone—caused lower inhibition (38.96 ± 0.91%). Sesamin (compound **2**), which is characterized by methylenedioxy rings between C3 and C4, induced 37.18 ± 0.47% inhibition of *α*-amylase activity, and the opening of the methylenedioxy ring (compound **6**) reduced this inhibitory effect (24.64 ± 1.35%). The presence of the glucose moiety at C3 increased the inhibitory activity of compound **7** to 45.36 ± 0.71%, whereas *β*-sitosterol caused inhibition of 13.78 ± 0.87%. Pentyl ferulate and 1-heneicosanol inhibited *α*- amylase activity by 19.47 ± 0.5 and 29.17 ± 0.97%, respectively. Acarbose was used as a reference drug and caused 97.98 ± 0.58% inhibition of *α*-amylase activity (Figure 5). 

##### Inhibitory Effect on Glycogen Phosphorylase

**Figure 6:**
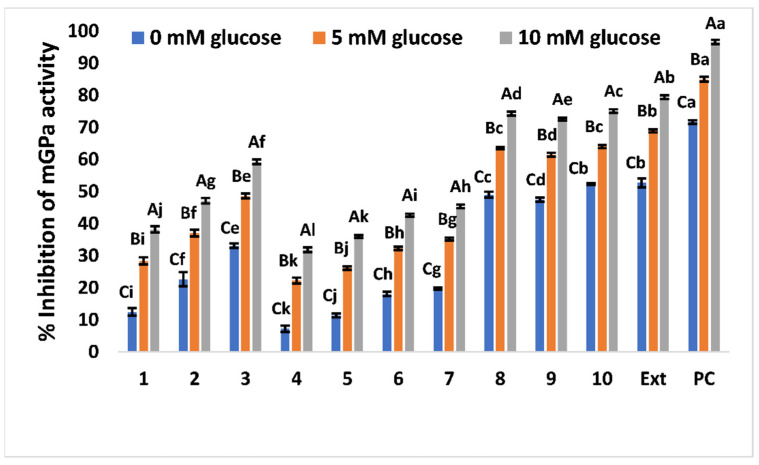
Effect of *G*. *abyssinica* extract (Ext) and isolated bioactive compounds on the glycogen phosphorylase activity. The activity of phosphorylated glycogen phosphorylase from rabbit muscle (mGPa) was measured, and CP-91149 was used as a positive control (PC). Lowercase letters indicate significant differences in mGPa activity among treatment conditions (*p <* 0.05). Upper-case letters indicate significant differences in mGPa activity among glucose concentrations (*p <* 0.05). Data are presented as the means ± standard deviations.

*G. abyssinica* extract and isolated compounds at a concentration of 2 mg/mL significantly (*p* < 0.05) decreased mGPa activity (Figure 6). The inhibitory effect of all tested compounds was significantly lower than that of CP-91149 (3.13 mM) (Figure 6). *G. abyssinica* extract exhibited the highest inhibitory activity, whereas the inhibitory ability of the isolated compounds decreased in the following order; **10** > **8** > **9** > **3** for different glucose concentrations (Figure 6). Additionally, the inhibitory effect on mGPa activity was regulated by the concentrations of glucose; specifically, the glucose concentration was directly proportional to the inhibition of mGPa activity (Figure 6).

##### Effects on Glucose Diffusion

**Figure 7:**
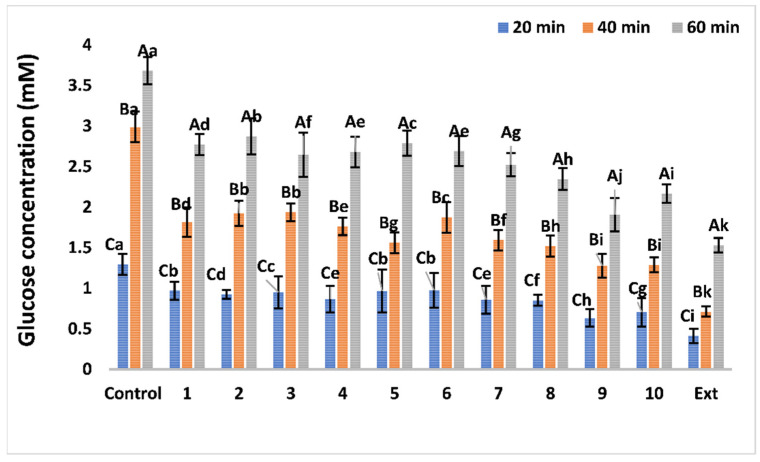
Effect of *G. abyssinica* extract and isolated bioactive compounds on the rate of glucose diffusion. Lower-case letters indicate significant differences (*p <* 0.05) between treatment conditions and upper-case letters indicate significant differences (*p <* 0.05) between time conditions. Data are presented as the means ± standard deviations.

The effect of the *G. abyssinica* extract and isolated compounds on the rate of glucose diffusion into an external solution was investigated (Figure 7). Isolated *G. abyssinica* compounds and methanolic extract at a concentration of 2 mg/mL caused a significant (*p* < 0.05) reduction in the diffusion rates of glucose measured after 20, 40, and 60 min compared with those obtained for the control (Figure 7). The decrease in glucose diffusion at different times was significantly greater (*p <* 0.05) in the presence of *G. abyssinica* extract than that measured with isolated compounds (Figure 7). Glucose diffusion across the dialysis membrane was directly proportional to time. In the presence of flavonoid glycosides (compounds **8**–**10**), glucose diffusion was significantly reduced, and the external glucose concentrations were 1.287–1.763 mM after 40 min. This represented a 49.18–57.22% decrease in the glucose movement (*p* ˂ 0.05) compared with that in the control (Figure 7). Moreover, the presence of a methoxy (-OMe) group at C4` in the B ring (compound **9**) slightly enhanced the ability to decrease glucose diffusion compared with those of compound **8** or **10**, while the high-molecular-weight flavonoid (compound **8**) did not affect the glucose diffusion rates which increased after 60 min compared with those obtained with compounds **9** and **10**. The rate of glucose diffusion into an external solution was overall lower than that of the control for all isolated *G. abyssinica* compounds and the extract. The effect of *G. abyssinica* extract and isolated compounds on the glucose concentration assay was evaluated to ensure that it produced valid results suitable for its intended purpose (Figure S37). The accuracy of the glucose concentration assay was not adversely affected by any of the isolated compounds or the *G. abyssinica* extract (Figure S37).

##### Effects on Glucose Uptake by Yeast Cells

**Figure 8:**
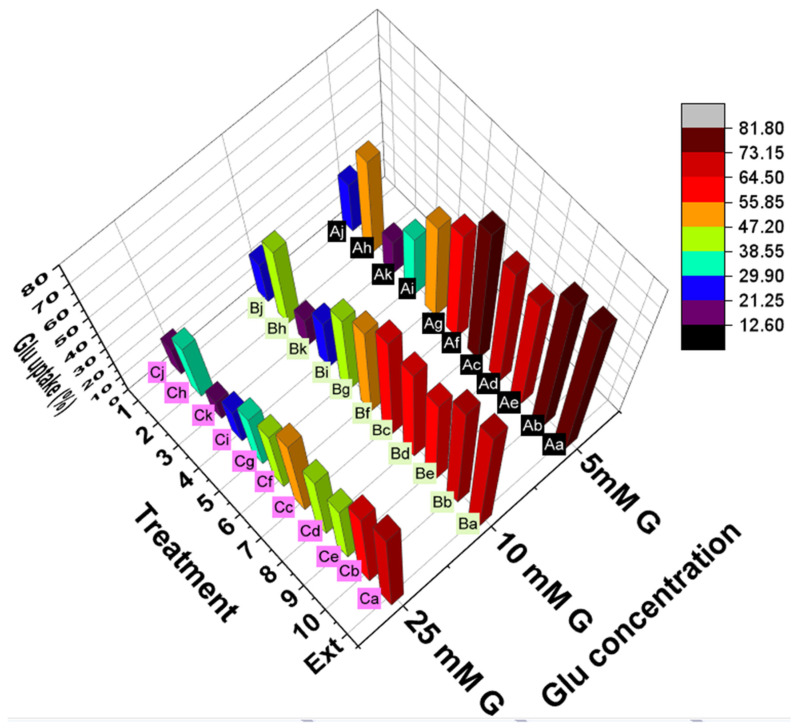
Glucose uptake by yeast cells exposed to different concentrations of glucose (5, 10, and 25 mM) in the presence of *G. abyssinica* extract or compounds 1–10. Lower-case letters indicate significant differences (*p <* 0.05) between treatment conditions and upper-case letters indicate significant differences (*p <* 0.05) between glucose concentrations.

The stimulation of glucose uptake by cells is a proposed mechanism responsible for the hypoglycemic effects of phytochemicals. This uptake was tested in vitro using the facilitated glucose diffusion in a yeast cell model. *G. abyssinica* extract and each compound stimulated glucose uptake through the plasma membrane of yeast cells (Figure 8). The rate of glucose uptake was inversely proportional to the molar concentration of glucose (Figure 8). Furthermore, a lower glucose concentration in the solution was associated with higher uptake by yeast cells. The highest glucose uptake by yeast cells was recorded for *G. abyssinica* extract in the presence of 5 mM glucose. For the same glucose concentration, the uptake decreased progressively in compounds **7**, **8**, and **10**, in descending order (Figure 8). The presence of the glucose moiety at the C3 of *β*-sitosterol 3-*O*-*β*-D-glucoside (**7**) increased the rate of glucose uptake by yeast cells to 75.97 ± 0.95% in the presence of 5 mM glucose compared with that obtained for **3** (60.96 ± 1.5%) with the same glucose concentration. The glucose uptake by yeast cells in the presence of 5 mM glucose was 68.75 to 80.15% after treatment with flavonoid glycosides (**8**–**10**), whereas the flavone genkwanin decreased glucose uptake to 53.04 ± 1.43% (Figure 8). The presence of the C4`-OMe group in the B ring of **9**, significantly (*p* ˂ 0.05) affected the glucose uptake by yeast cells to 65.29 ± 0.57% in the presence of 5 mM glucose compared with that obtained after treatment with **8** and 10 (80.14 ± 0.59 and 68.75 ± 0.95%, respectively). 

### 3.4. PCA of the Multifunctional Activity of G. abyssinica Extract and Isolated Compounds

**Figure 9:**
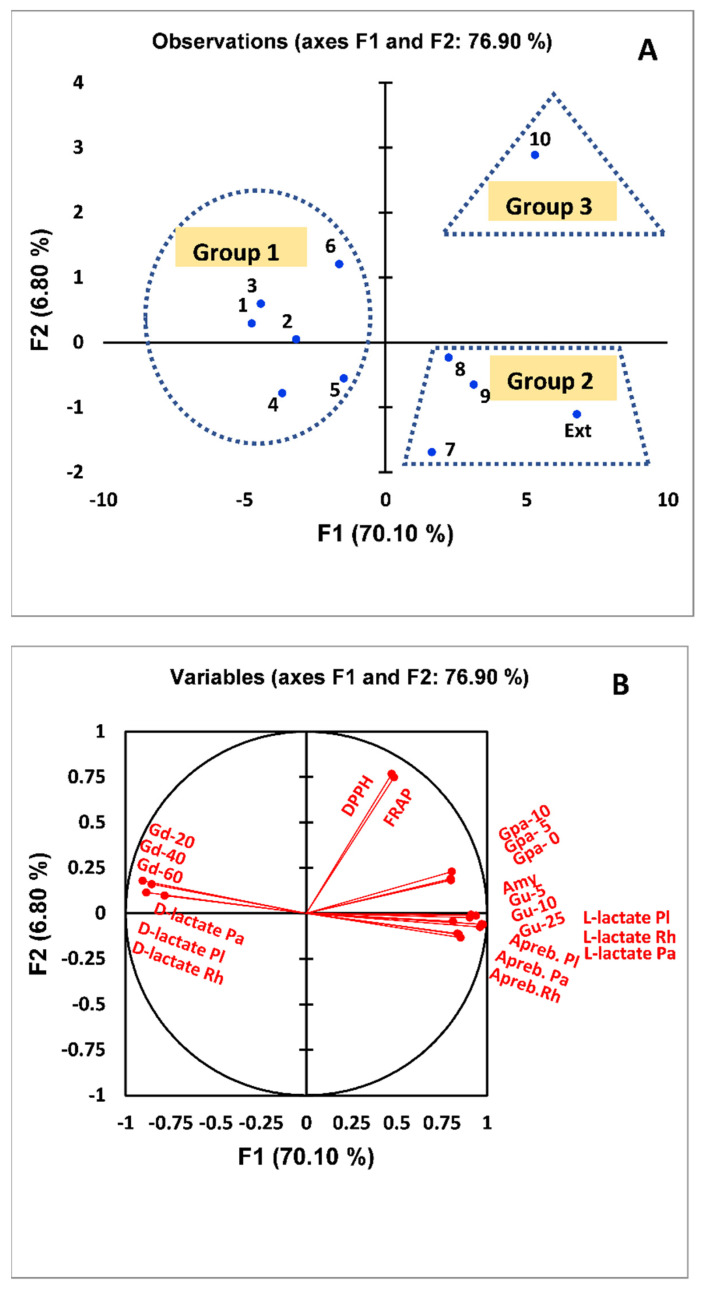
(**A**) Biplot of the principal component analysis (PCA) of the impact of the *G. abyssinica* extract and isolated bioactive compounds on the prebiotic activity score (A_preb_), D-(−)-lactic acid and L-(+)-lactic acid levels, antioxidant activity (DPPH and FRAP assays), glucose uptake by yeast cells (Gu), inhibition of *α*-amylase (Amy) and glycogen phosphorylase (Gpa) activities, and glucose diffusion (Gd). (**B**) Analysis of the correlations among variables.

The PCA of antidiabetic, antioxidant, and prebiotic activities of *G. abyssinica* extract and isolated compounds using two PCs explained 76.90% of the variability (Figure 9). PC1 (70.10%) comprised all tested parameters except for the antioxidant activity (FRAP and DPPH assays). PC2 (6.80%) included the FRAP and DPPH analysis data (Figure 9). Four groups were identified using PCA: Group 1 was positioned on the negative side of PC1, whereas groups 2 and 3 were on the positive side of PC1. 

Group 1 was characterized by higher glucose diffusion rates at different times and higher D-(−)-lactic acid concentrations in the different *Lactobacillus* strains. Group 2 typically had higher prebiotic activity scores (A_preb_*)* and L-(+)-lactic acid concentrations in all strains tested, glucose uptake by yeast cells, and inhibition of *α*-amylase activity (Figure 9A). The hallmarks of Group 3 were higher antioxidant activity and an inhibitory effect on the glycogen phosphorylase activity (Figure 9A). Partial least-squares regression plays a critical role when many explanatory variables are correlated with an effect. This statistical assay was performed to assess the relationships between the prebiotic (A_preb_ and optically active form of lactic acid), antioxidant, and antidiabetic activities. The effective inhibition of *α*-amylase and glycogen phosphorylase was positively correlated with the A_preb,_ L-(+)-lactic acid concentration, antioxidant properties measured by DPPH and FRAP, and glucose uptake by yeast cells (Figure 9B), whereas it was negatively correlated with the concentration of D-(−)-lactic acid and rate of glucose diffusion (Figure 9B). Furthermore, glucose diffusion was positively correlated with the D-(−)-lactic acid concentration (Figure 9B).

## 4. Discussion

Although glucose is generally utilized as a source of carbon for the cultivation of strains of Lactobacilli, their ability to use different isolated compounds is well-established and acknowledged to depend on the strain [35]. 

Our findings led to the conclusion that the tested probiotic strains were successful in using all of the isolated compounds and *G. abyssinica* extract. A similar dependency was observed by Elbermawi et al [31] in terms of the extracts of *Rumex vesicarius* causing greater rates of growth compared with the isolated phenolic compounds, except for *β*-sitosterol. Our results indicate that the presence of a glucose moiety C3 of the C-ring for flavonoids (**8**–**10**) and *β*-sitosterol 3-*O*-*β*-D-glucoside increased the prebiotic activity score sharply compared with the other phenolic substances that were examined in this investigation. The presence of a glycosyl-conjugated group on phenolic compounds may improve the growth of probiotic bacteria by providing fermentable sugars [36,37]. This might explain the growth-promoting influences of the glycosylated compounds **7-10** on the tested probiotic strains [36,37]. Contrary to the data presented in this study, the presence of the glucose moiety at C3 decreased the prebiotic activity score of *β*-sitosterol 3-*O*-*β*-D-glucoside compared with *β*-sitosterol when used *E. coli* Nissle 1917 [31]. Many studies were concerned with combining both probiotic bacterial families, such as *Lactobacillaceae* and *Bifidobacteriaceae*, and phenolic compounds [38]. Many polyphenols and their metabolites improve the growth of probiotics in the human intestinal tract [38].

Regarding the enzymatic transformation of phenolic compound, *Lb. plantarum* is unquestionably the best-reported probiotic species [38]. *Lb. plantarum* can produce benzyl alcohol dehydrogenase, which reduces NAD^+^ and reversibly oxidizes certain aromatic alcohols to aldehydes [38]. *Lb. plantarum* also has the ability to hydrolyze non-volatile, odorless glycosides through aglycones’ formation by glycosidase [38]. Phenolic acid decarboxylase allows *Lb. plantarum* to hydrolyze ferulic acid, caffeic acid, and p-coumaric acid. *Lb. plantarum* may degrade gallic acid esters’ ester linkages and tannins because it can produce tannin acyl hydrolase [38]. Decarboxylases, reductases, and esterases are some of the other enzymes that *Lactobacillus* bacteria can synthesize [39]. 

Generally, the two optical isomers (L(+) and D(−)) are formed due to the fermentation of a carbon source. The type of carbon and the bacterial strain, however, can affect the ratio of these optical forms [27,40], which is consistent with the present results. The body may be harmed by the accumulation of D-lactic acid because it may cause D-lactic acidosis, which can cause behavioral abnormalities, disorientation, ataxia, or even comas [27,40]. L-lactic acid acts as a chemical that delivers energy to neurons, but the D-lactate isomer may decrease its availability for neurons [41]; for this reason, it can be deemed advantageous for L-lactic acid production to increase at the expense of D-lactic acid concentration caused by the carbon source. To the best of our knowledge, no research has been performed on the impact of polyphenols as a carbon source on the racemic mixture of both lactic acid isomers produced by lactic acid bacteria.

Moreover, it was observed that the isolated compounds analyzed in the current study did not influence the activities of the tested enzymes of the probiotic *Lactobacillus* spp. strains. Common enzyme activity for *Lactobacillus* spp. was seen, regardless of the nature of the compounds. For instance, *β*-galactosidase causes the hydrolysis of lactose, and acid phosphatase helps to obtain energy from phosphates; naphthol-AS-BI-phosphohydrolase dephosphorylates proteins, alkaloids, and nucleotides; and *β*-glucosidase breaks glycosidic bonds and tolerates high temperatures [42,43]. None of the probiotic strains studied possess any enzymes that could be harmful, such as chymotrypsin, which is linked to gastrointestinal tract diseases, and *β*-glucuronidase, which has carcinogenic, mutagenic, and toxic potential [42,44]. However, cystine arylamidase, alkaline phosphatase, and n-acetyl-*β*-glucosaminidase activities were carbon-source-dependent. Compounds **1**, **3** and **4** inactivate the alkaline phosphatase in *Lb. rhamnosus* and *Lb. plantarum*, while in *Lb. paracasei*,this enzyme is inactivated by **2** and **6**. alkaline phosphatase inactivation is detrimental to the strains’ probiotic qualities, because this enzyme could reduce inflammatory responses and encourage immunomodulation in the gastrointestinal tract [45]. Furthermoe, it was observed that compounds from **1** to **6** increased the activity of *n*-acetyl-*β*-glucosaminidase, which is an adverse effect, because this enzyme is involved in intestinal diseases [44]. 

Flavonoids (**5** and **8**–**10**) displayed significant antioxidant activity, particularly in the FRAP assay. The hydroxylation degree and configuration of the B ring were the most significant factors affecting the scavenging of different radicals, because one hydrogen and electron are given to radicals of various types, resulting in the formation of stable radicals. Flavonols are phenolic compounds with strong antioxidant activity, as established both experimentally and theoretically [46]. In the present study, the flavonol glycoside **10** displayed the highest antioxidant activity. Overall, the 2,3-double bond with the 4-oxo group, which provides conjugation, and the 3-hydroxyl group, which forms hydrogen bonds with ring (B) hydroxyls, afford flavonols their potent antiradical activity [47]. It is well known that flavonols with a 3`,4`-catechol structure in the B ring are highly active radical scavengers due to the formation of a stable ortho-semiquinone in the B ring [46]. In compound **10**, ring B contained one additional hydroxyl group (pyrogallol group) that further increased the antioxidant capacity. Theoretically, the glycosylation of flavonols at the C3-hydroxyl group diminishes their radical scavenging capacity. However, our study showed that **10**, a flavonol glycoside, had the best antioxidant activity. Similarly, compound **10** was characterized by Hayder, et al [48] as a very strong radical scavenger with an extremely low IC_50_ value of 1.4 µg/mL using the DPPH assay. Interestingly, the computational analysis conducted by Mendes, et al [49] to explore the antioxidant potential in the gas status predicted that compound **10** had an antioxidant capacity similar to that of its aglycon. They concluded that the lowest bond dissociation enthalpy for **10** (74.72 kcal/mol against 74.8 kcal/mol) was also the lowest for the OH groups in other compounds. Flavonoid compounds **5**, **8**, and **9** had lower antioxidant activity than compound **10**, likely because of the difference in the oxygenation pattern. 

A broad and diverse class of polyphenols known as lignans is formed from phenylpropanoid dimers in which the central carbon of their propyl side chains links C6–C3 units. Furofuran lignans containing a 2,6-diaryl-3,7-dioxabicyclo[3.3.0]octane skeleton constitute one of the main subclasses of the lignan family of natural compounds. Lignans bearing two benzylic groups (4-hydroxy-3-methoxyphenyl) directly linked to the furan ring, such as pinoresinol, have an antiradical activity against DPPH-like ferulic acid [50]. It has been reported by Eklund, et al [51] that the radical scavenging capacity of lignans containing catechol-(3,4-dihydroxyphenyl) moieties is quite high, whereas the comparable guaiacyl-(3-methoxy-4-hydroxyphenyl) lignan-like molecules (**6**) had a somewhat reduced scavenging potential. The antioxidant capability has been previously reported for **6** showing a moderate antioxidant activity with an IC_50_ of 137.6 μM [52], compound **6** might exert its antiradical activity by donating one hydrogen atom from its guaiacyl moiety, resulting in the dimerization of two lignan radicals. The kinetics of the reaction between DPPH and some furofuran lignans have been investigated by Eklund et al [51]. This study showed that, following intramolecular hydrogen transfer, the principal termination reaction, which includes the radical coupling at position 5, produces dimeric structures. Higher oxidation at the benzylic position in lignans has been associated with decreased antioxidant efficacy [50]. Similarly, Nakai, et al [53] concluded that DPPH^•^, hydroxyl (^•^OH), and super-oxide anion (O_2_^•−^) radicals were seldom scavenged or inhibited by sesamin. Wan, et al [54] reported that the sesamin has poor antioxidant activity in vitro, but a noticeably great antioxidant capacity in vivo.

The urgent need to find antidiabetic drugs is constantly increasing. As *α*-amylase is a crucial step in the digestion of polysaccharides and regulates glucose postprandial blood levels, it is a potential functional target for the treatment of T2D [55]. Furthermore, the *α*-amylase inhibitory activity of ethanolic extract of *G. abyssinica* leaves has been reported with an IC_50_ of 75 µg/mL [17].

The most active flavonoid was compound **10**. It has been reported that the hydroxylation of the B ring enhances flavonoids’ abilities to suppress *α*-amylase activity [56,57,58]. An -OH group at C7 of the A ring and C4` of the B ring enhanced the inhibitory properties of compounds **8** and **10** than those of **5** and **9** [55]. 

The inhibition of *α*-amylase activity induced by compound **9** was lower than that of **8** and **10**, indicating that the methoxyl substitution diminished the inhibitory effect of flavonoids on *α*-amylase. Proença et al [55], revealed that the methoxylation and methylation of flavonoids reduce their ability to inhibit *α*-amylase and this finding is in a full agreement with our results. 

Glycogen phosphorylase is a key enzyme that causes hyperglycemia by degrading glycogen. Therefore, using glycogen phosphorylase inhibitors in high-glucose-level conditions observed in T2D contributes to treating diabetic patients [59]. Flavonoids are considered the most important glycogen phosphorylase inhibitors [60]. However, their binding sites and their application as inhibitors of glycogen phosphorylase have not been fully explored [59]. The comparison of the inhibitory effect of compound **5** (contains a substituted hydroxyl at C7) with those of compounds **8**-**10** (free C7 hydroxyl group), indicated that the hydroxyl substituent at C7 enhanced the mGPa inhibition. Therefore, the inhibitory effect of flavonoids on glycogen phosphorylase activity is stimulated by the addition of a hydroxy substituent at the C5 and C7 of the A ring and the C3`, C4`, and C5` of the B ring, such as in compound **10**. Consequently, the B ring hydroxylation and the presence of substituents in the B and C rings are important for the flavonoid to act as inhibitors [61,62].

The present study confirmed the significance of *G. abyssinica* extract or isolated compounds in combating diabetes, primarily by lowering glucose absorption. Further research into the therapeutic application of these extracts or chemicals is necessary to determine their impact in delaying or preventing diabetes complications. Our data are consistent with those of Sattar, et al [63], they reported that ginger extracts reduce or prevent glucose diffusion, with the high concentrations of extract (20 and 40 g/L) reducing glucose diffusion the most (32 to 46.7% reduction, respectively), and the main mode of action of extracts or nutrients for slowing down glucose diffusion and delaying the digestion and absorption of carbohydrates relies on the viscosity of the extracts or nutrients, which bind with glucose and consequently lower the concentration of glucose available in the small intestine [63]. Gallagher et al [64] investigated the ability of various plants to decrease the diffusion rates of glucose and reported that the highest decrease in glucose diffusion was caused by avocado and agrimony (more than 60%). Coriander, juniper, mushrooms, eucalyptus, mistletoe, and lucerne also markedly reduced glucose diffusion (ranging from 6 to 48%), whereas elder and nettle extracts did not affect the amount of glucose moving into external solutions. However, Büyükbalci and El [65] found that the overall glucose diffusion in the presence of ten aqueous herbal tea extracts was higher than that in the control. 

Regarding the *G. abyssinica* extract, the observed effect on the reduction in glucose diffusion throughout the study time can be attributed to the presence of macromolecules, such as polysaccharides, present in the extract, as it has been reported that particles can form a physical barrier to the movement of glucose through entrapping glucose within the matrix [32,34,66]. However, the reduction in glucose diffusion caused by the isolated compounds (**1**–**10**) can be attributed to, but not limited to, the adsorption of the isolated phenolic compounds by the cellulose membrane (dialysis bag with a cutoff of 500–1000). The complex formation between purified compounds and cellulose (dialysis membrane) represents a physical barrier to the movement of glucose molecules and glucose entrapment within a complex matrix. Hydrogen bonds and hydrophobic interactions are the two main driving forces proposed to explain the nature of complex formations between polyphenols and cellulose [67,68]. The amount of polyphenols that are bound to cellulose membranes increases in direct proportion to the molecular weight of the polyphenols [68]. The number of aromatic moieties and hydroxyl groups present in the molecular structures, or the native charge of phenolic compounds, are potentially important factors for the non-covalent formation of hydrogen bonds and hydrophobic interactions between polyphenols and cellulose [68,69]. Tang, et al [70] reported that the phenolic compounds with more aromatic rings in their molecular structure tend to bind more strongly to cellulose than compounds with a single aromatic ring. A significant finding of the previous studies is that neutrally charged cellulose interacts with negatively charged phenolic acids, positively charged anthocyanins, and neutral compound (+/−)-catechin with comparable rates and extents. This further supports the hypothesis that non-covalent interactions are mainly responsible for the associations of polyphenols with dietary fiber, as has been previously proposed [71,72]. In agreement with our hypothesis, Padayachee, et al [73] also found that either positively charged anthocyanins or negatively charged phenolic acids in purple carrot juice interacted with bacterial cellulose analogues to comparable extents. 

Generally, glucose transporter molecules in the cell membrane ensure glucose uptake by skeletal muscles. When the blood insulin levels are high, myocytes and/or leptocytes control these transporters, inducing hypoglycemia [74]. In contrast, studies focusing on how medications affect postprandial hyperglycemia have been key to the management of diabetes mellitus, and decreasing postprandial hyperglycemia is a therapeutic strategy that has received a lot of attention to date. 

Moreover, attention has been drawn to the mechanism of glucose transport across the yeast cell membrane as a crucial technique for the in vitro testing of the hypoglycemic effects of various compounds/medical plants [34,74,75,76]. However, the consumption of glucose by human body cells or other eukaryotic cells is possibly distinct from that of yeast cells. The transport of glucose across yeast cell membranes may involve facilitated diffusion, rather than using the phosphotransferase enzyme system as a mediator [74]. The transport of glucose across membranes in yeast may be affected by many variables, including the concentration and metabolism of glucose inside the yeast cells. If most of the internal glucose is transformed into other metabolites, the intracellular concentration of glucose will decrease and the uptake of glucose into the cell will increase. Likewise, the transport of glucose across the yeast cell membrane in the presence of *G. abyssinica* extract or isolated compounds might implicate increased glucose metabolism and facilitate diffusion. Investigating the activity of *G. abyssinica* extract and isolated compounds characterized in the present study in vivo would unquestionably be interesting and might contribute to improving the uptake of glucose by adipose tissues and muscle cells of the human body. *G. abyssinica* extract or isolated compounds might bind glucose effectively, allowing glucose transport through the cell membrane for further metabolism [74].

## 5. Conclusions

The extract of *G. abyssinica* and isolated metabolites decreased the activities of *α*-amylase, glycogen phosphorylase and the glucose diffusion, but they improved the yeast cell absorption of glucose. Furthermore, the extract and isolated compounds showed antioxidant activities in DPPH and FRAP assays. They promoted the growth of *Lactobacillus* strains, increased concentration of L- (+)-lactic acid, rather than D- (−)-lactic acid and had no negative effects on their enzymatic profile. The present study revealed that the plant *G. abyssinica* is rich in bioactive compounds that may be used to treat diabetes and enhance the gastrointestinal tract functionality and health; however, further in vivo studies are still required. 

## Figures and Tables

**Table 1:** Antioxidant activities of *G. abyssinica* extract and isolated compounds in two different antioxidant assays.

Compounds	Antioxidant Activity in FRAP Assay	Antioxidant Activity in DPPH Assay
	(µmol TE/g Compound or Extract)	(µmol TE/g Compound or Extract)
**1**	492 ± 4.58 ^e^	192.67 ± 5.03 ^d^
**2**	122.33 ± 1.53 ^gh^	24.45 ± 0.80 ^g^
**3**	131.67 ± 5.69 ^g^	12.67 ± 1.16 ^h^
**4**	105.67 ± 3.5 ^h^	7.36 ± 0.95 ^h^
**5**	694.26 ± 5.45 ^d^	107.57 ± 3.5 ^e^
**6**	2119.33 ± 6.03 ^b^	237 ± 3.0 ^c^
**7**	149 ± 7.55 ^f^	18.55 ± 0.94 ^g^
**8**	508.67 ± 8.02 ^e^	108.84 ± 3.05 ^e^
**9**	688 ± 6.57 ^d^	63.74 ± 3.2 ^f^
**10**	2666.67 ± 7.5 ^a^	4635.33 ± 6.02 ^a^
***G. abyssinica*** **extract**	1059 ± 4.58 ^c^	378 ± 7.9 ^b^

The antioxidant activity was expressed in micromoles of Trolox equivalent (TE) per gram of dried extract or compound. The data represent the means ± standard deviations of three independent measurements (*n* = 3). ^a–h^ Superscripts indicate values that were statistically different (*p* ˂ 0.05) from that obtained with the control (Trolox). DPPH: 2,2-diphenylpicrylhydrazyl (DPPH) and FRAP: ferric-reducing antioxidant power (FRAP) assays.

## Data Availability

All of the data is contained within the article and the Supplementary Materials.

## Supplementary Materials

The following supporting information can be downloaded at: https://www.mdpi.com/article/10.3390/antiox11122482/s1, Figures S1–S36: One-dimensional and two-dimensional NMR spectra (1H, APT, HSQC, and HMBC) and HRESIMS for compounds **1**–**10** and Figure S37: The effect of *G. abyssinica* extract and isolated compounds on accuracy of the glucose concentration assay are available in supplementary data.
