# Supplementary material for: In Vitro Antidiabetic, Antioxidant, and Prebiotic Activities of the Chemical Compounds Isolated from Guizotia abyssinica
Source: Antioxidants (Basel). 2022 Dec 16;11(12):2482. doi: 10.3390/antiox11122482 (PMC9774103; doi:10.3390/antiox11122482)
Supplement: Supplementary file 1 [file antioxidants-11-02482-s001.zip › antioxidants-2010981-supplementary.pdf]

Supplementary Materials

List of contents

Figure S1	HRESIMS spectrum of compound 1	
Figure S2	^{1}H-NMR spectrum of compound 1	
Figure S3	APT spectrum of compound 1	
Figure S4	HSQC spectrum of compound 1	
Figure S5	HMBC spectrum of compound 1	
Figure S6	^{1}H-NMR spectrum of compound 2	
Figure S7	APT spectrum of compound 2	
Figure S8	HSQC spectrum of compound 2	
Figure S9	HMBC spectrum of compound 2	
Figure S10	^{1}H-NMR spectrum of compound 3	
Figure S11	IR spectrum of compound 4 (GC-1)	
Figure S12	HRESIMS Mass spectrum of compound 4 (GC-1)	
Figure S13	^{1}H-NMR spectrum of compound 4 (GC 1)	
Figure S14	APT spectrum of compound 4 (GC1)	
Figure S15	HRESIMS Mass spectrum of compound 5 (GC-2)	
Figure S16	^{1}H-NMR spectrum of compound 5 (GC 2)	
Figure S17	APT spectrum of compound 5 (GC2)	
Figure S18	HMBC spectrum of compound 5	
Figure S19	Expansion of HMBC spectrum of compound 5 (GC2)	
Figure S20	HRESIMS Mass spectrum of compound 6 (GC-3)	
Figure S21	^{1}H-NMR spectrum of compound 6 (GC 3)	
Figure S22	APT spectrum of compound 6 (GC3)	
Figure S23	HSQC spectrum of compound 6	
Figure S24	HMBC spectrum of compound 6	
Figure S25	^{1}H-NMR spectrum of compound 7 (GC 4)	
Figure S26	APT spectrum of compound 7 (GC4)	
Figure S27	^{1}H-NMR spectrum of compound 8	
Figure S28	APT spectrum of compound 8	
Figure S29	HSQC spectrum of compound 8	
Figure S30	HMBC spectrum of compound 8	
Figure S31	^{1}H-NMR spectrum of compound 9	
Figure S32	APT spectrum of compound 9	

Supplementary Materials

Figure S33	HSQC spectrum of compound 9	
Figure S34	HMBC spectrum of compound 9	
Figure S35	^{1}H-NMR spectrum of compound 10	
Figure S36	APT spectrum of compound 10	
Figure S 37	The effect of *G. abyssinica* extract and isolated compounds on accuracy of the glucose concentration assay	

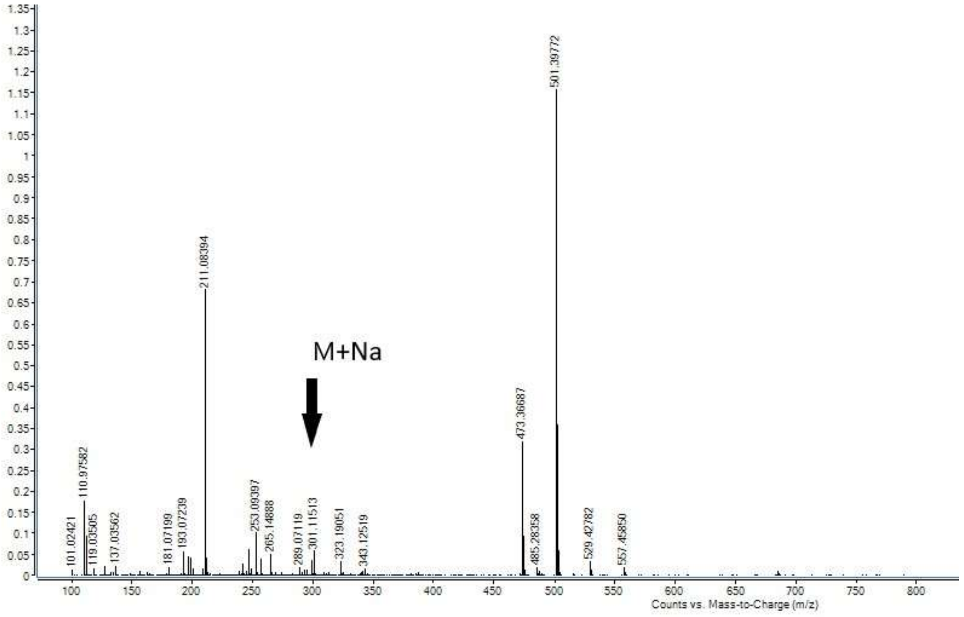

Figure S1: HRESIMS spectrum of compound 1

Supplementary Materials

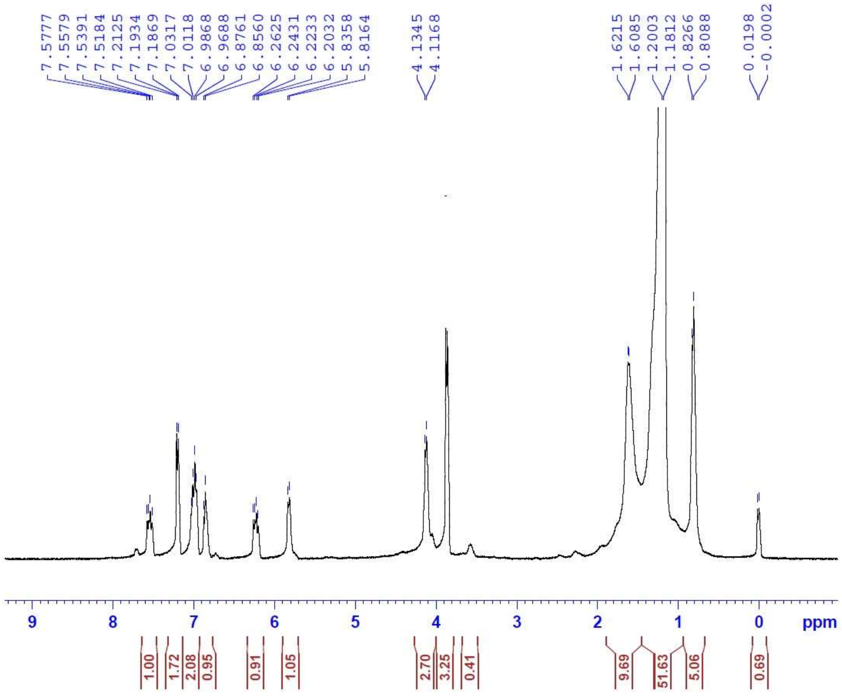

Figure S2: HNMR spectrum of compound GU2

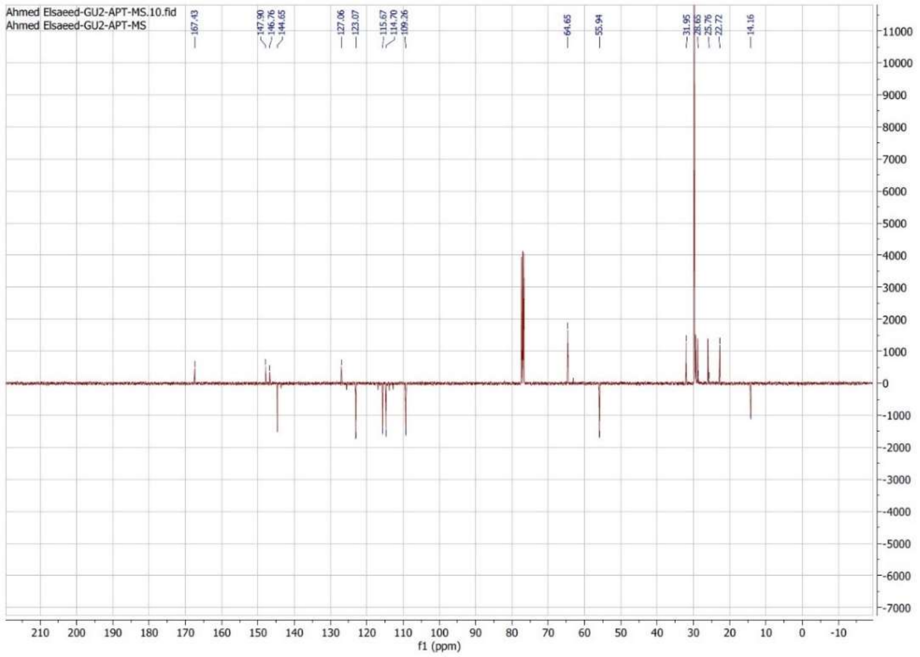

Figure S3: APT Spectrum of compound GU-2

Supplementary Materials

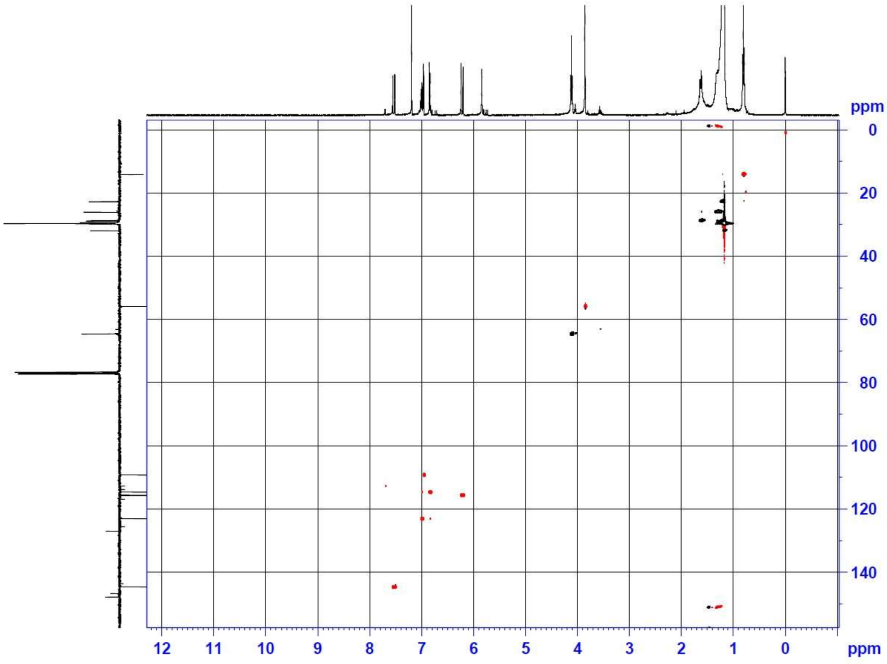

Figure S4: HSQC spectrum of compound GU2

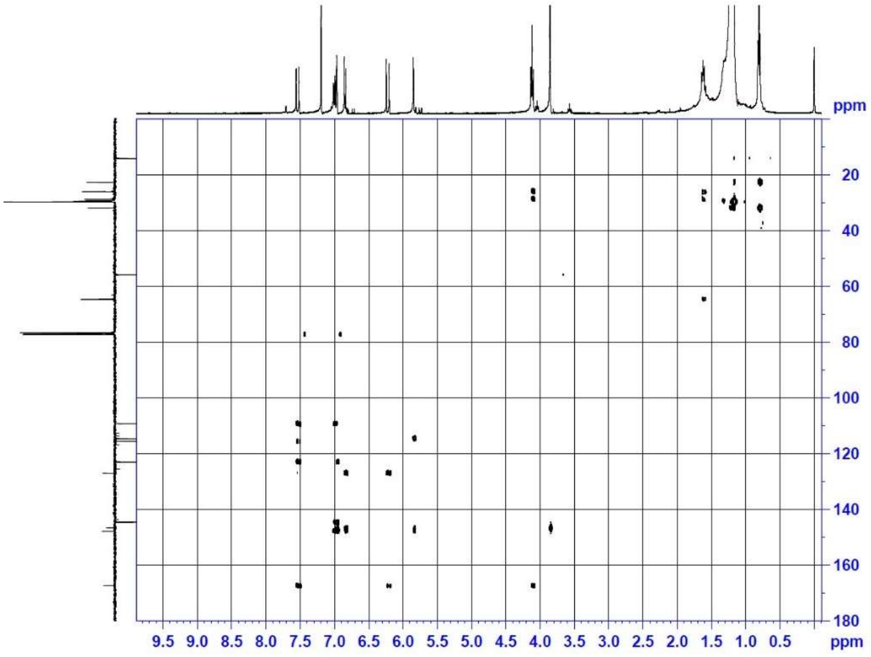

Figure S5: HMBC spectrum of compound GU2

<u>Supplementary Materials</u>

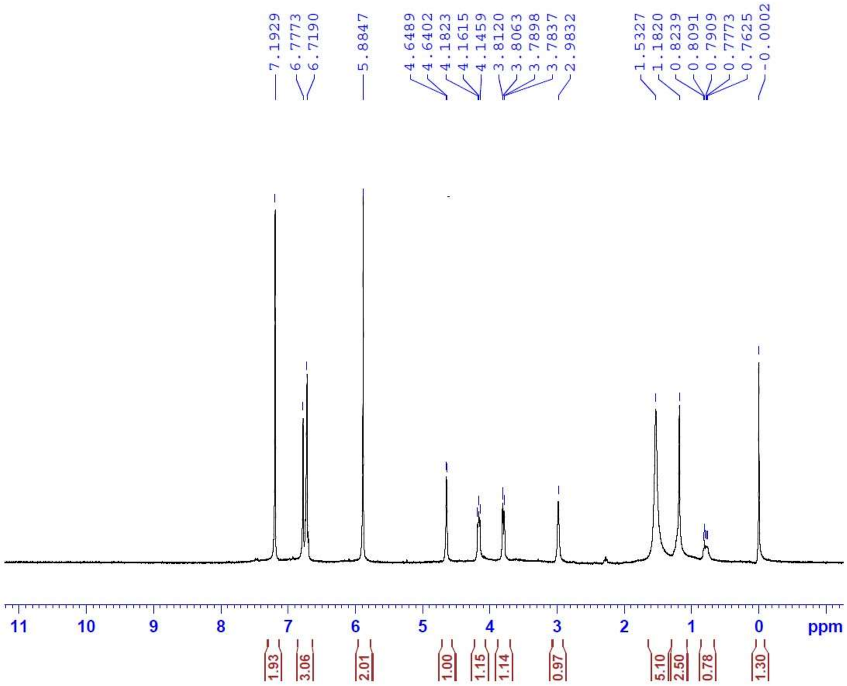

Figure S6: HNMR spectrum of compound GU3

Supplementary Materials

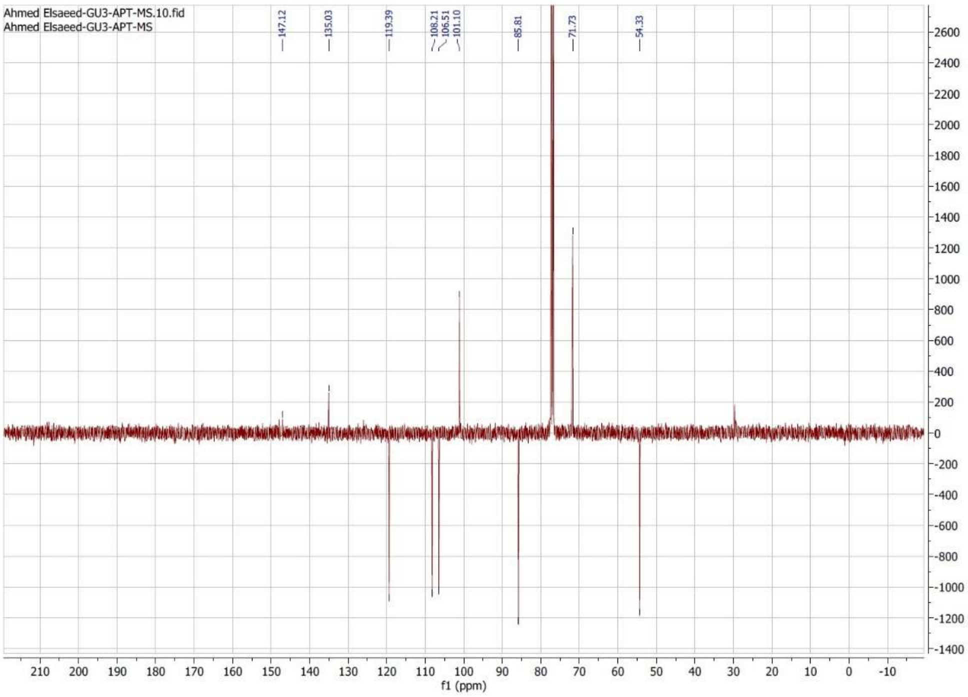

Figure S7: APT Spectrum of compound GU-3

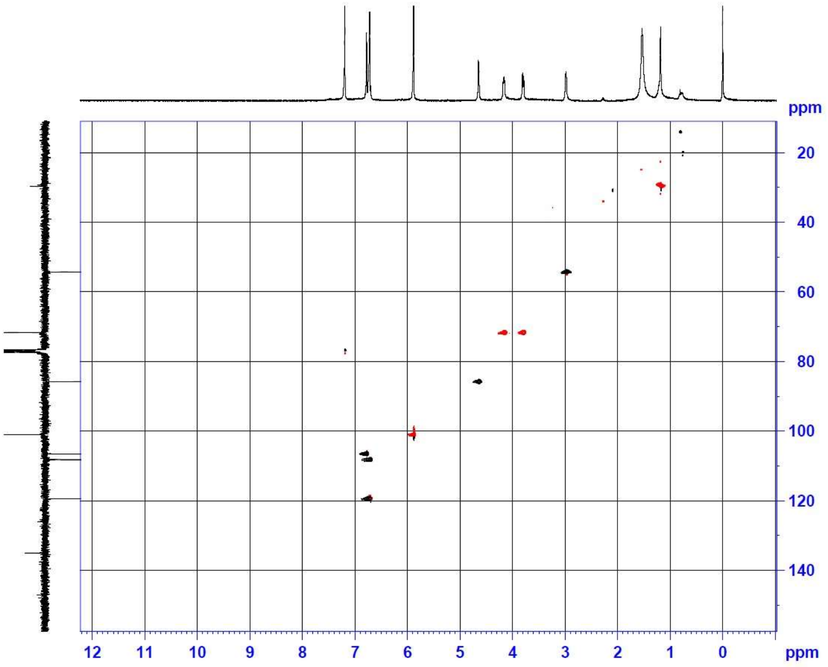

Figure S8: HSQC of compound GU-3

Supplementary Materials

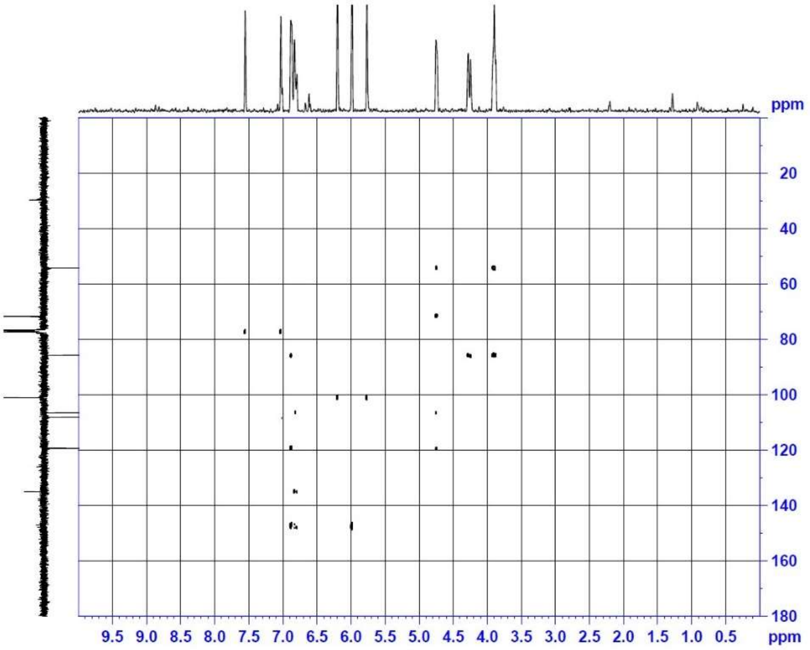

Figure S9: HMBC of compound GU-3

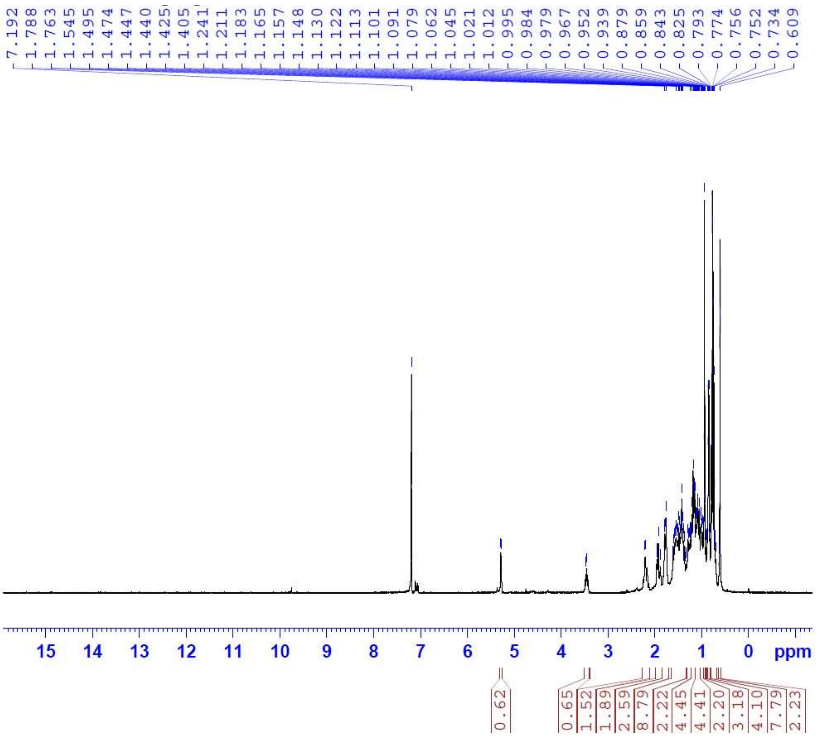

Figure S10: HNMR spectrum of compound 3

Supplementary Materials

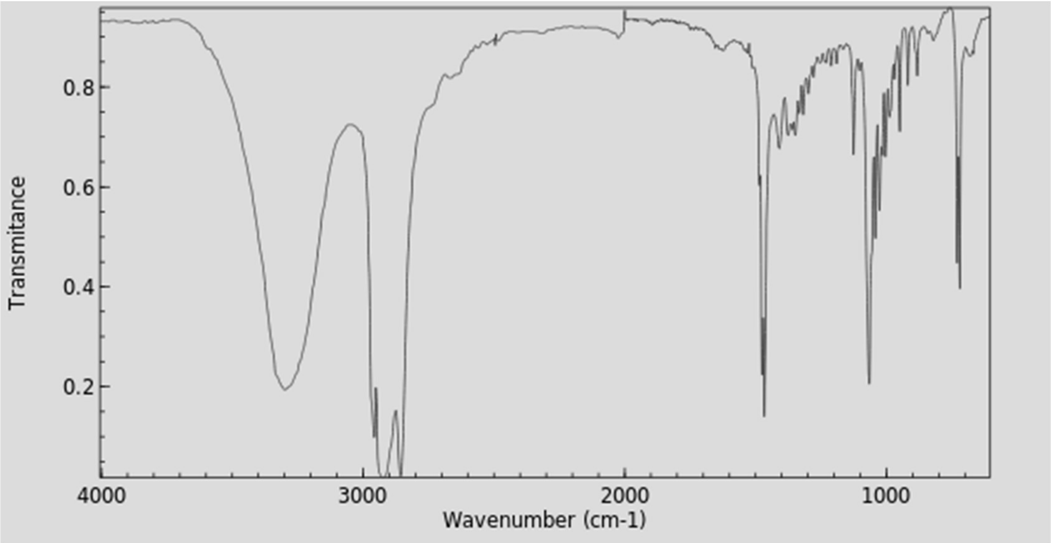

Figure S11: IR spectrum of compound 4 (GC-1)

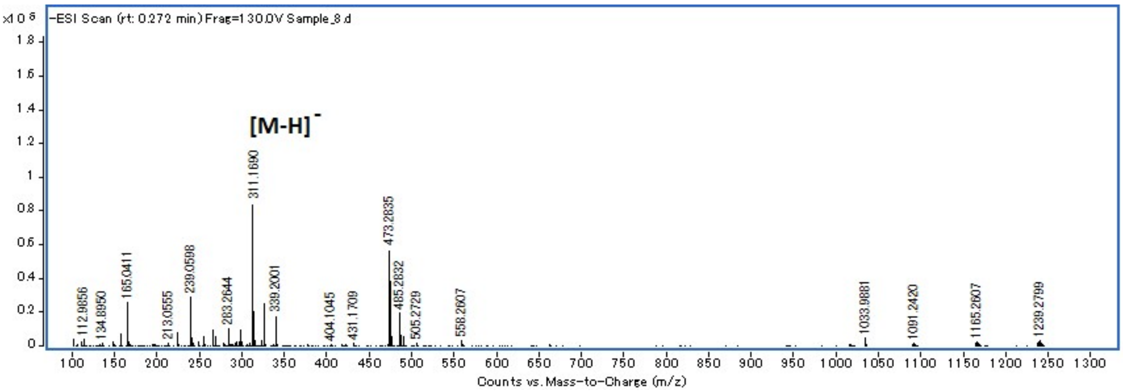

Figure S12: HRESIMS Mass spectrum of compound 4 (GC-1)

Supplementary Materials

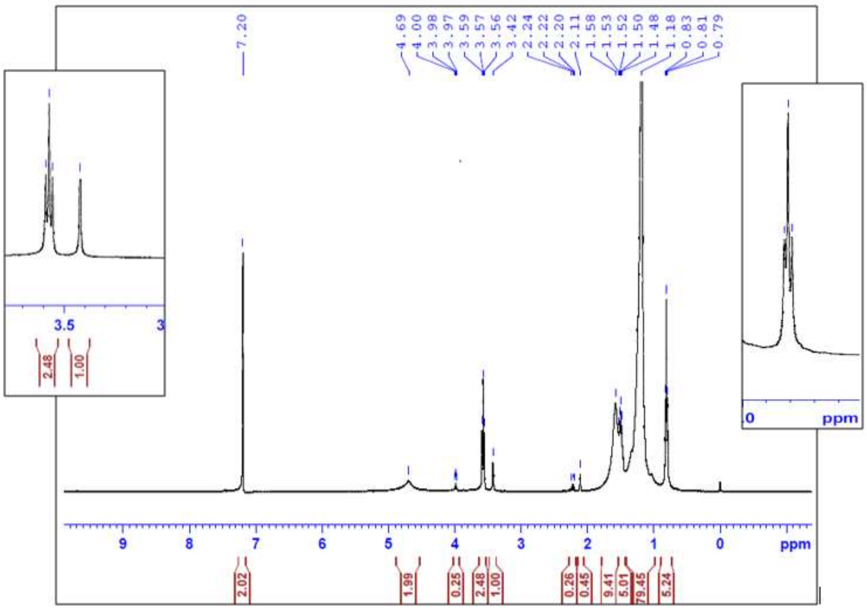

Figure S13: HNMR spectrum of compound 4 (GC 1)

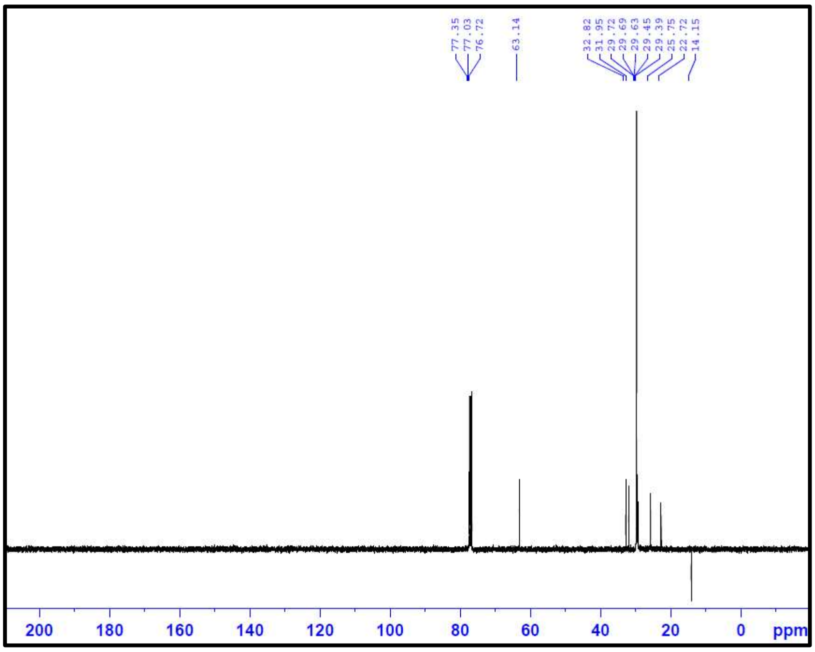

Figure S14: APT spectrum of compound 4 (GC1)

Supplementary Materials

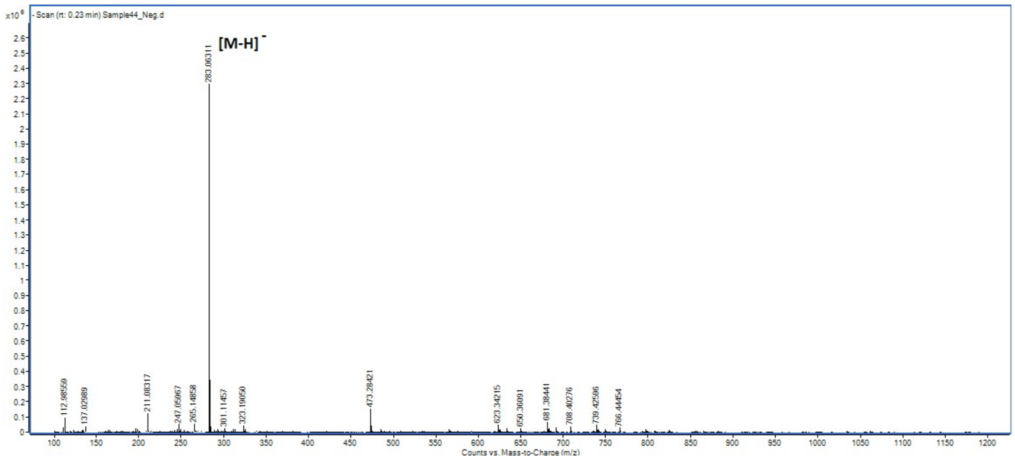

Figure S15: Mass spectrum of compound 5 (GC-2)

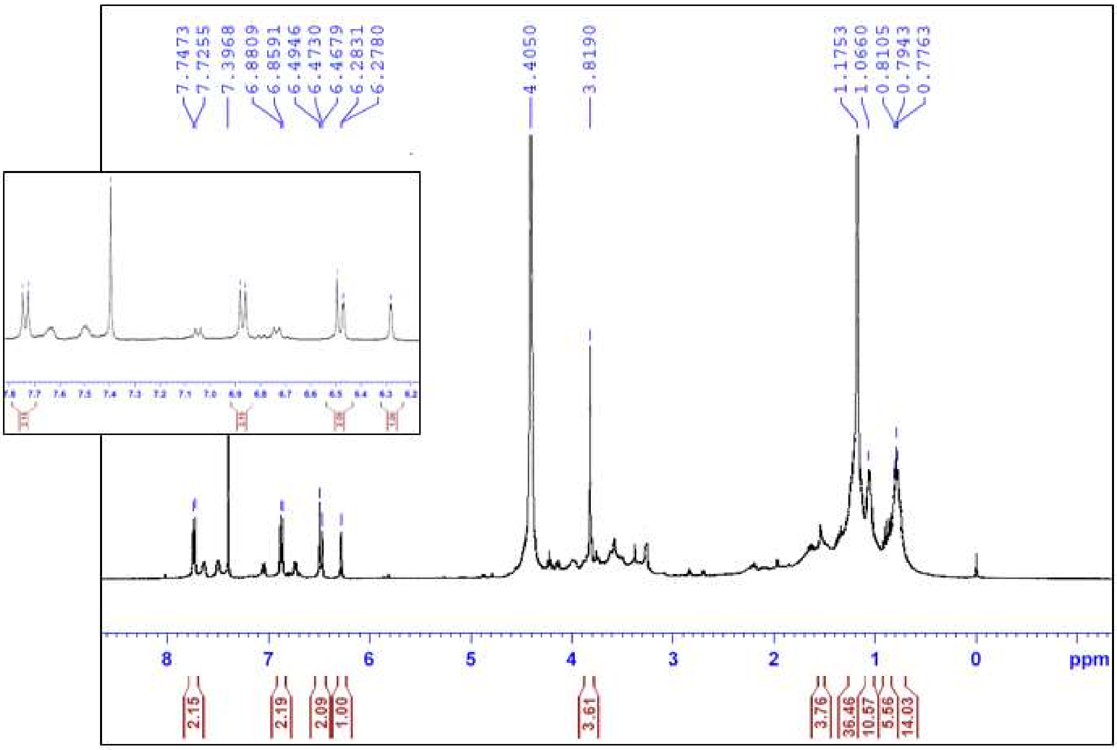

Figure S16: HNMR spectrum of compound 5 (GC 2)

Supplementary Materials

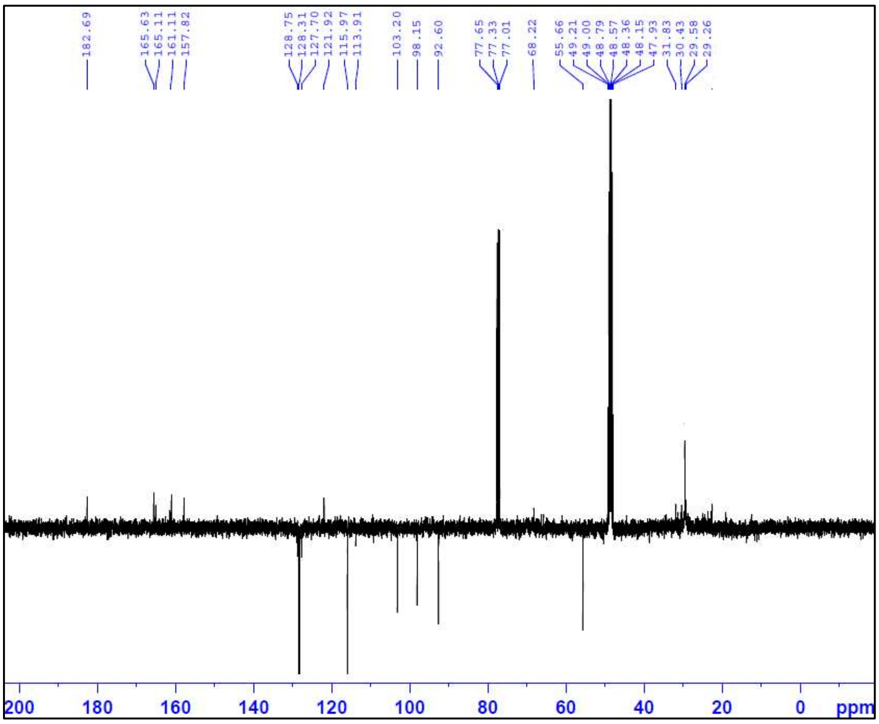

Figure S17: APT spectrum of compound 5 (GC2)

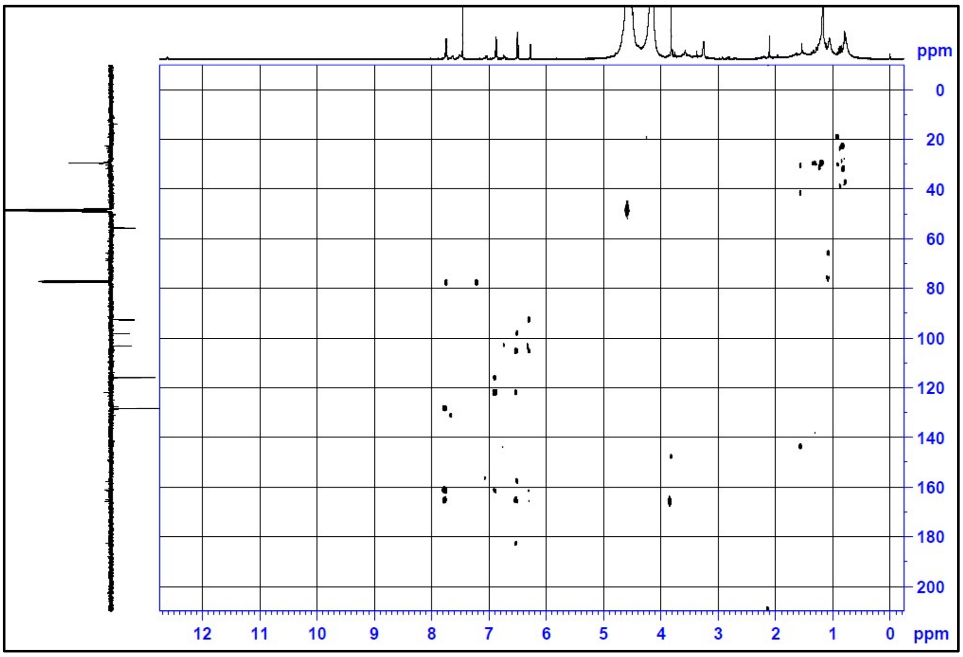

Figure S18: HMBC spectrum of compound 5 (GC2)

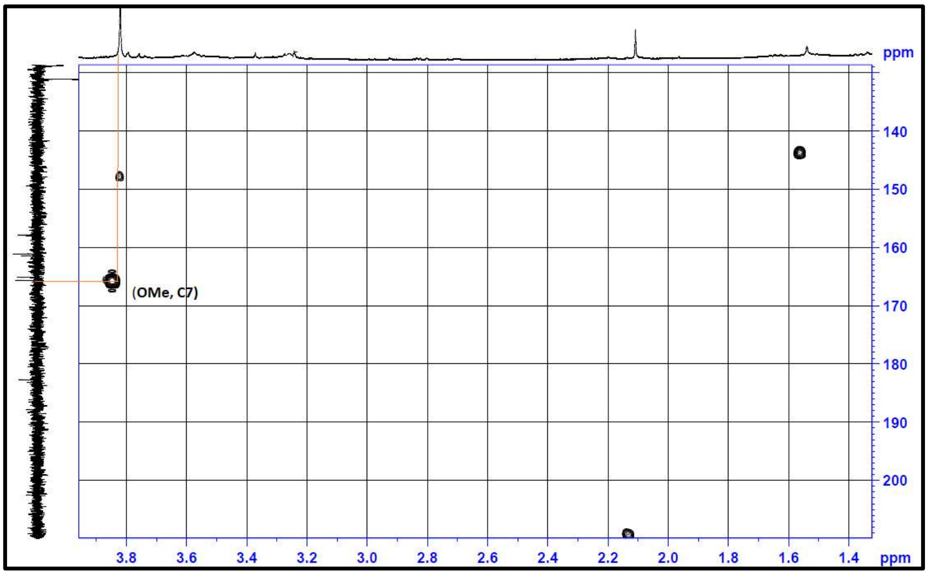

Figure S19: Expansion of HMBC spectrum of compound 5 (GC2)

Supplementary Materials

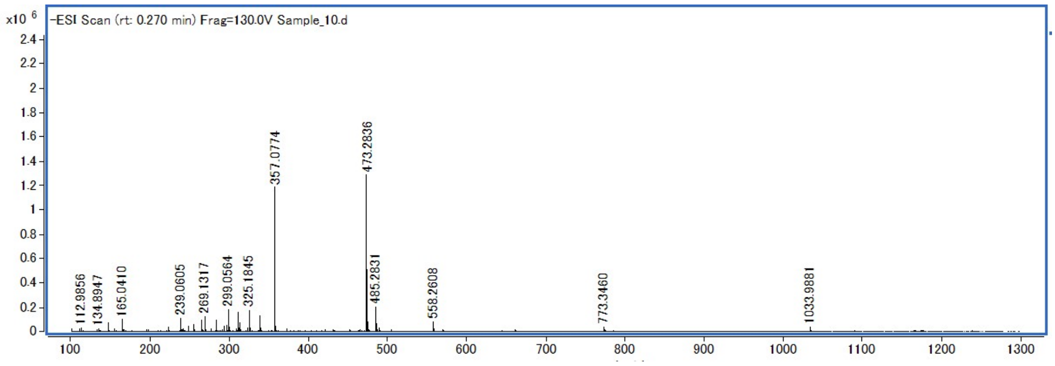

Figure S20: HRESIMS Mass spectrum of compound 6 (GC-3)

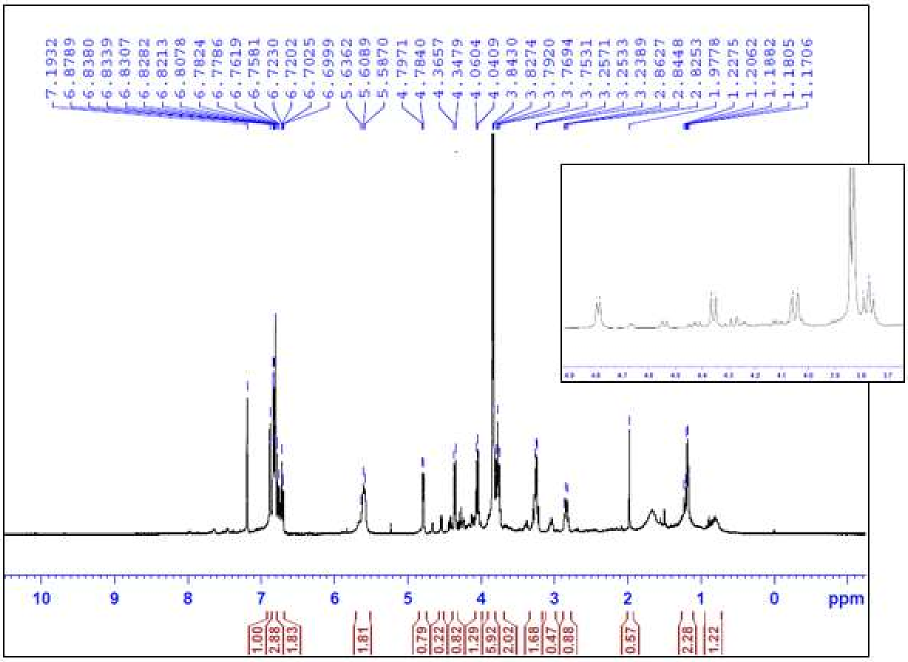

Figure S21: HNMR spectrum of compound 6 (GC 3)

Supplementary Materials

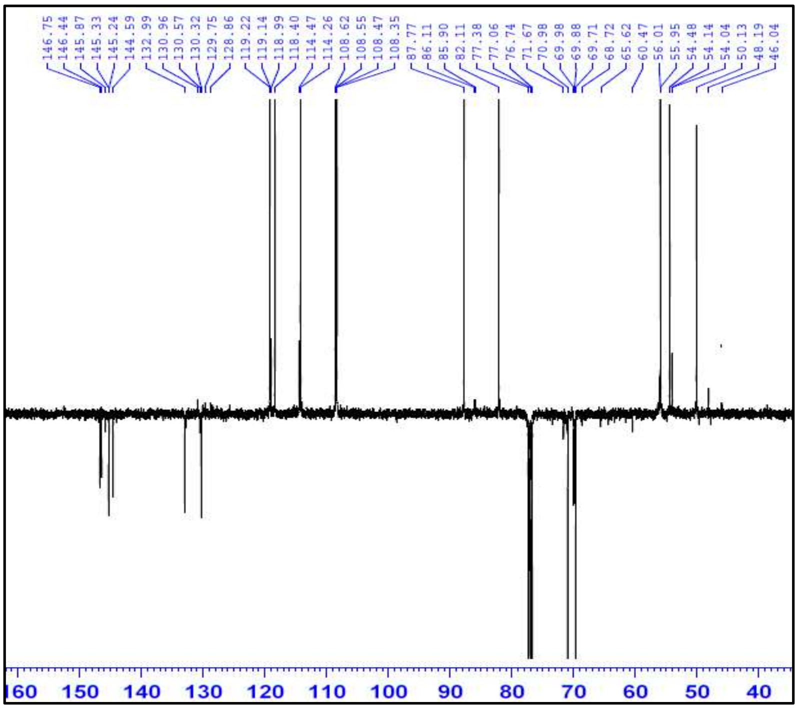

Figure S22: APT spectrum of compound 6 (GC 3)

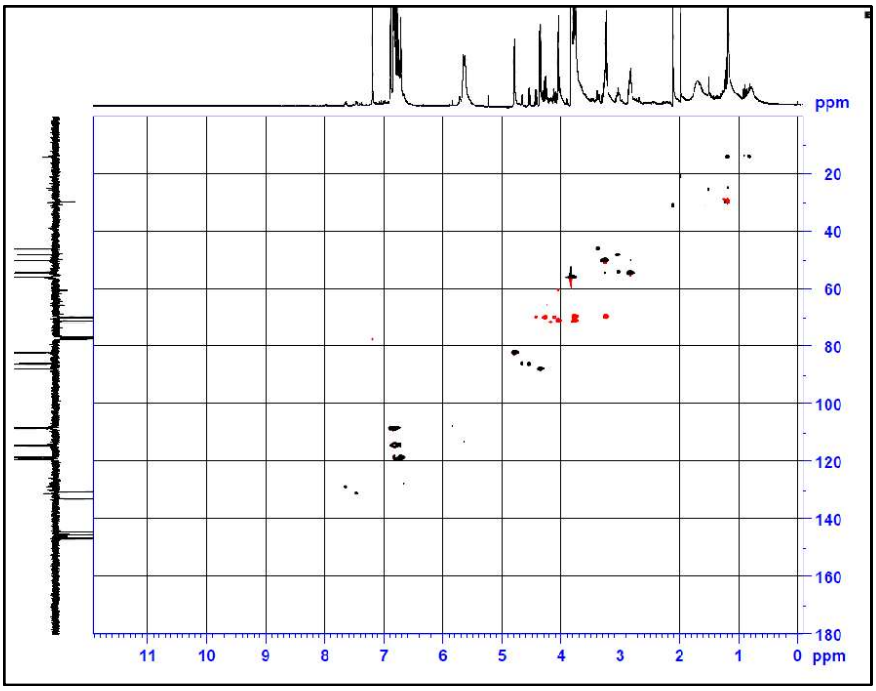

Figure S23: HSQC spectrum of compound 6 (GC 3)

Supplementary Materials

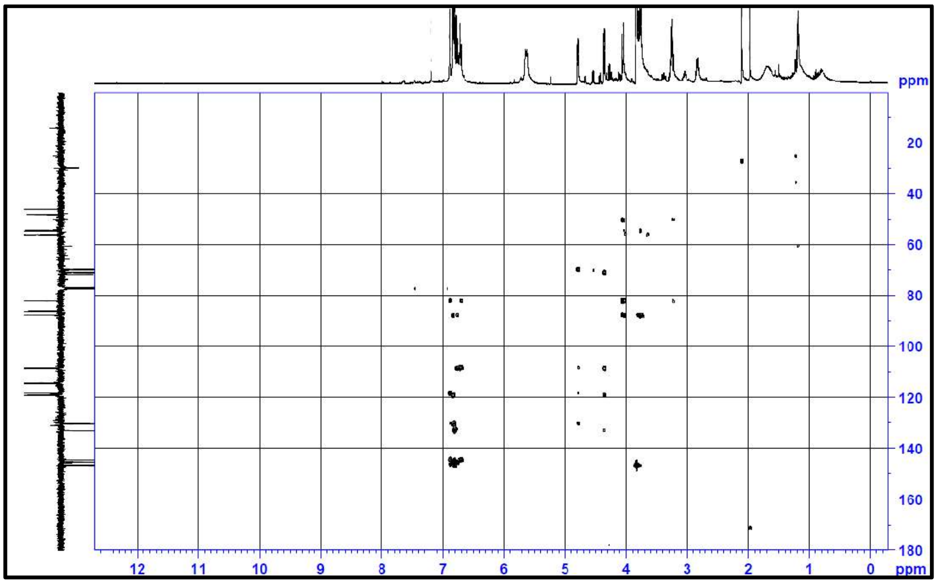

Figure S24: HMBC spectrum of compound 6 (GC 3)

Supplementary Materials

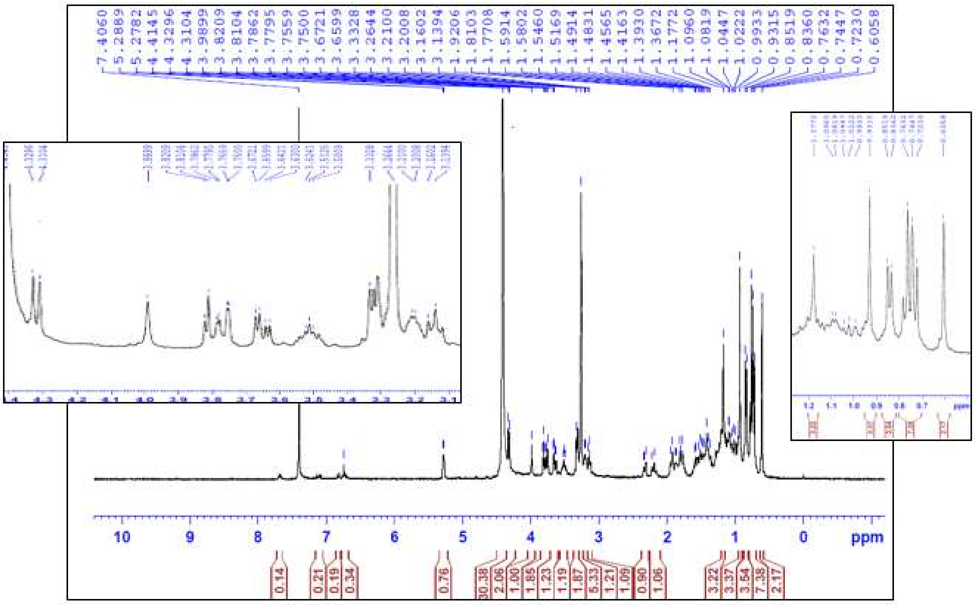

Figure S25: HNMR spectrum of compound 7 (GC 4)

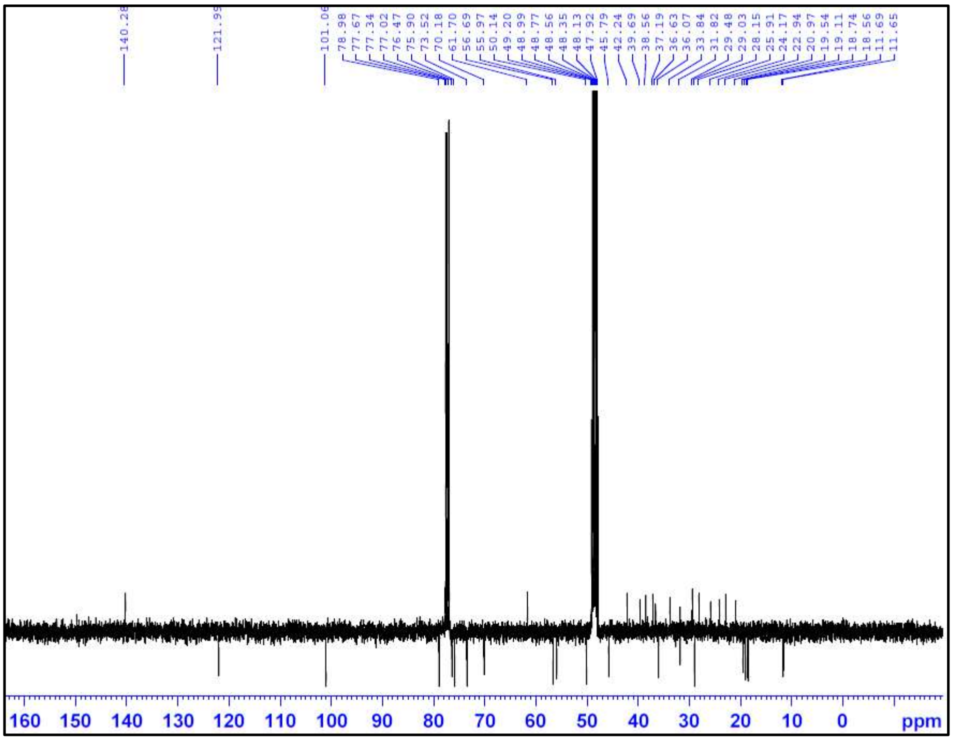

Figure S26: APT spectrum of compound 7 (GC 4)

Supplementary Materials

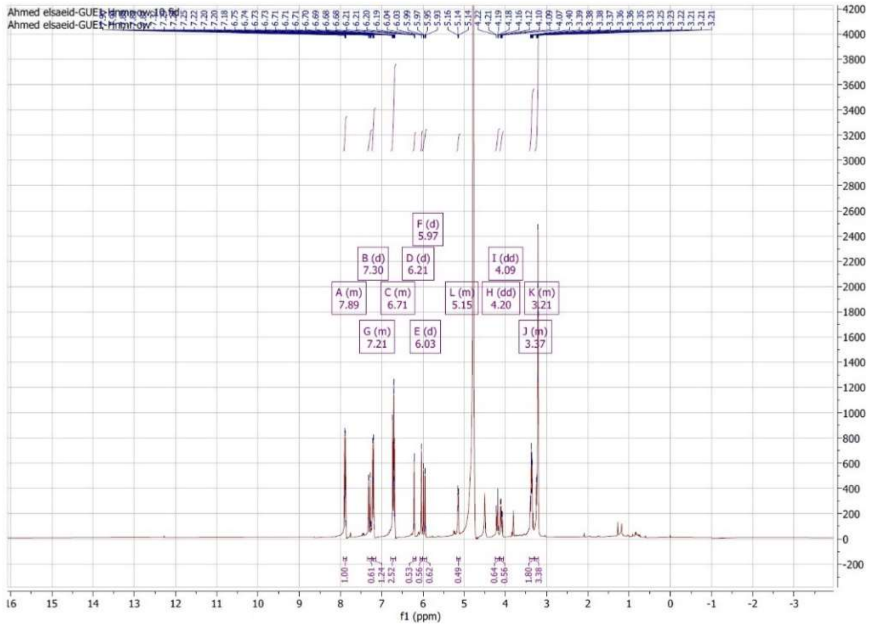

Figure S27: ^{1}H-NMR spectrum of compound 8 GUE-1

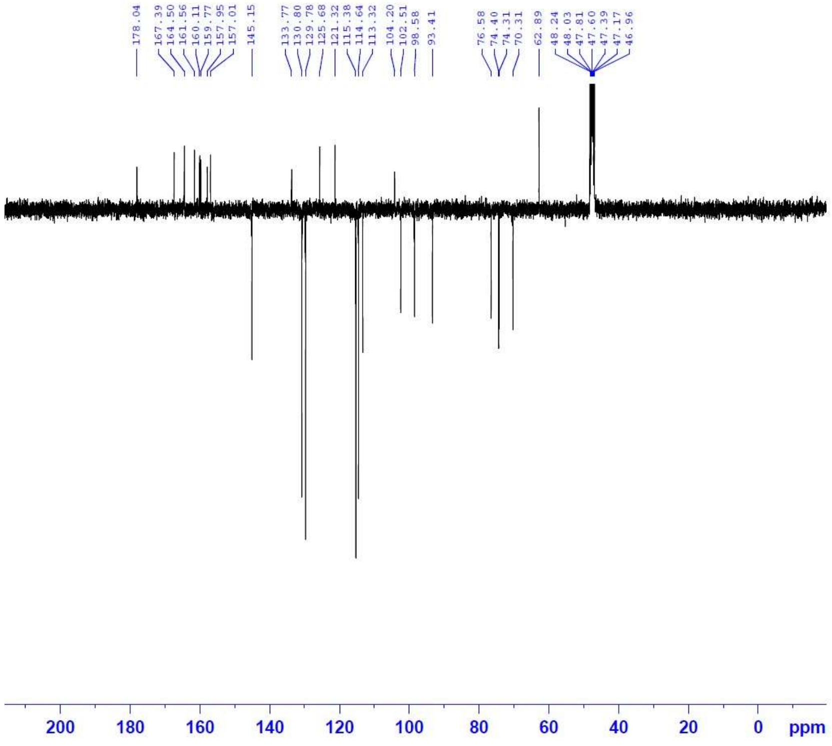

Figure S28: APT spectrum of compound 8 GUE-1

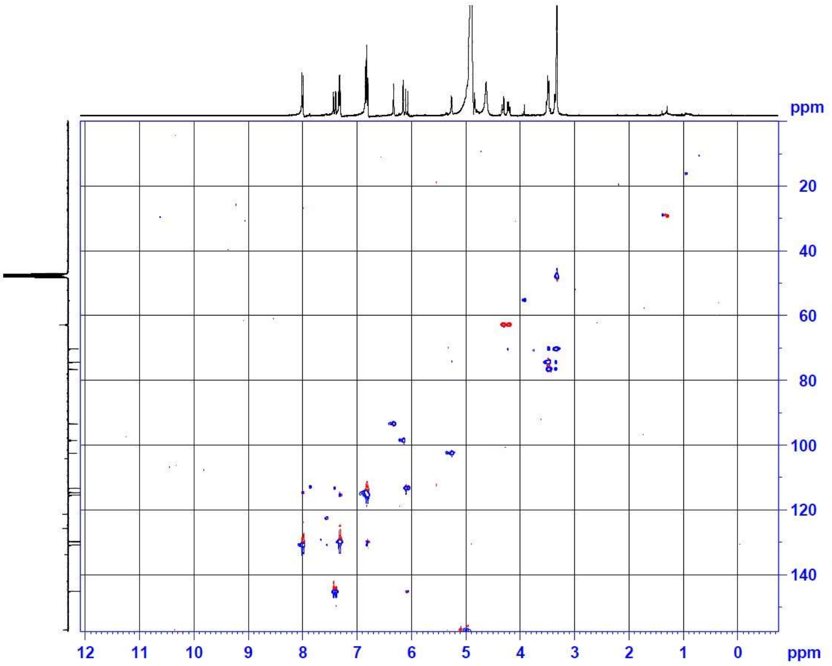

Figure S29: HSQC spectrum of compound 8 GUE-1

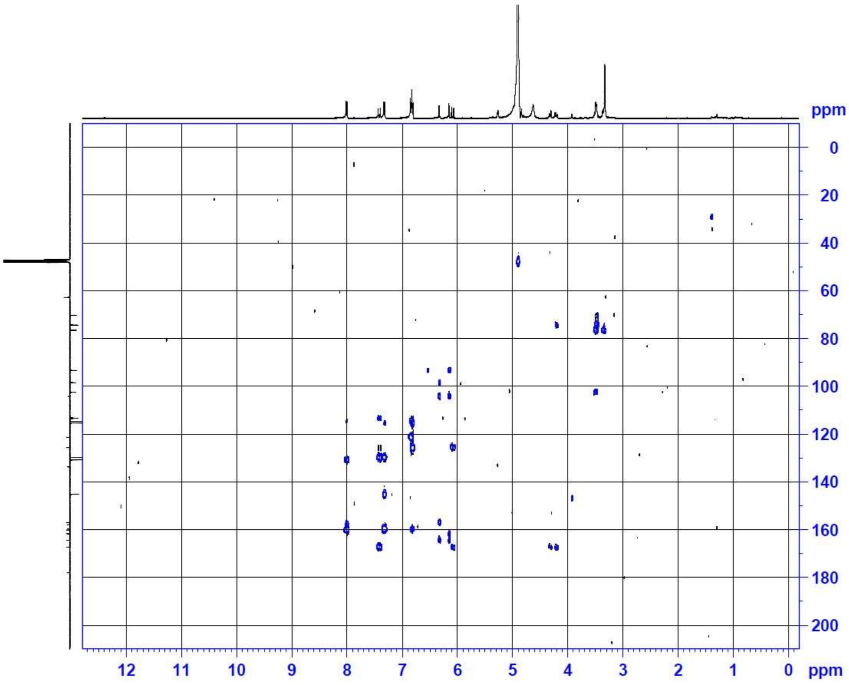

Figure S30: HMBC spectrum of compound 8 GUE-1

Supplementary Materials

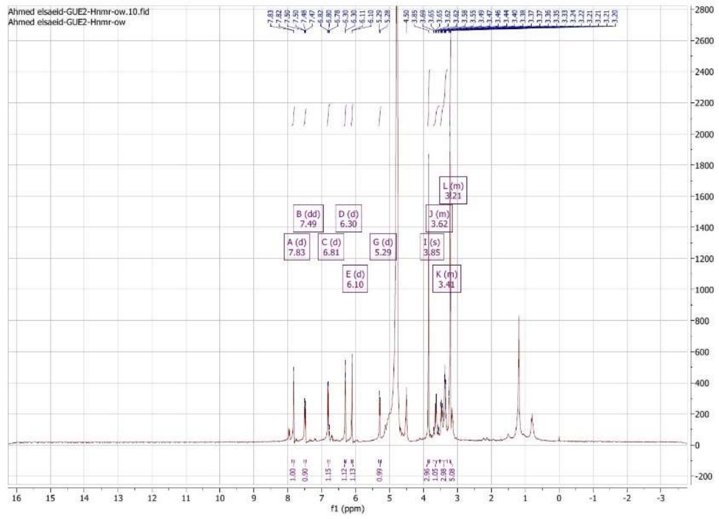

Figure S31: ^{1}H-NMR spectrum of compound 9 GUE2

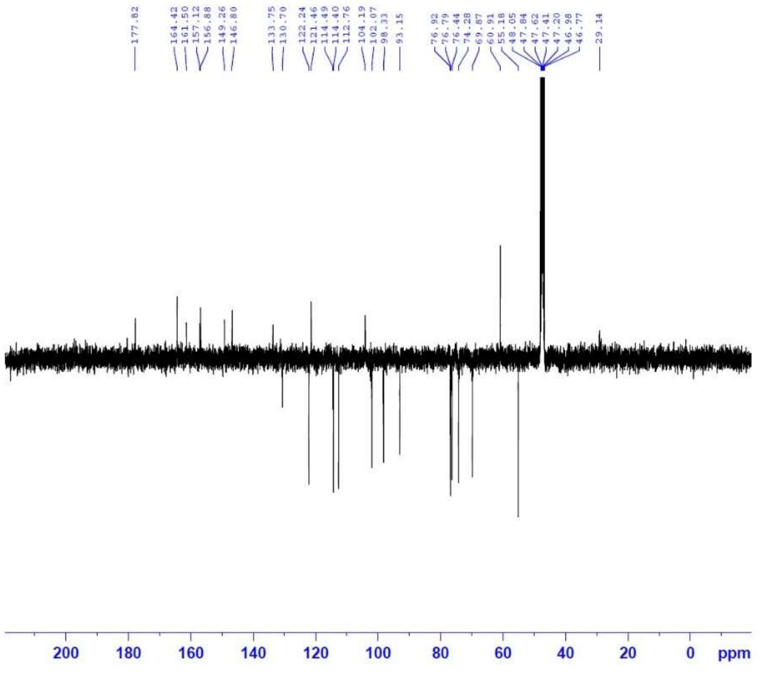

Figure S32: APT spectrum of compound 9 GUE2

Supplementary Materials

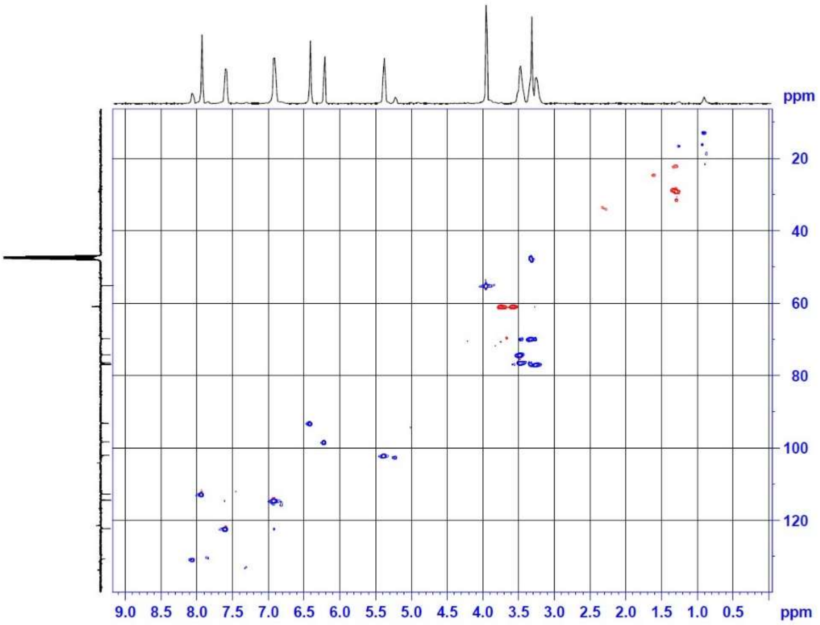

Figure S33: HSQC spectrum of compound 9 GUE2

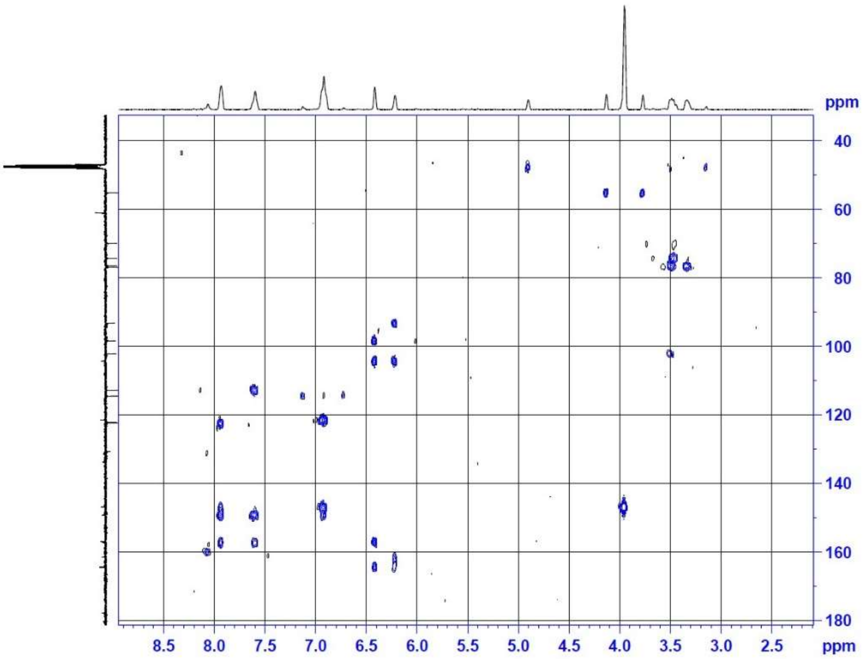

Figure S34: HMBC spectrum of compound 9 GUE2

Supplementary Materials

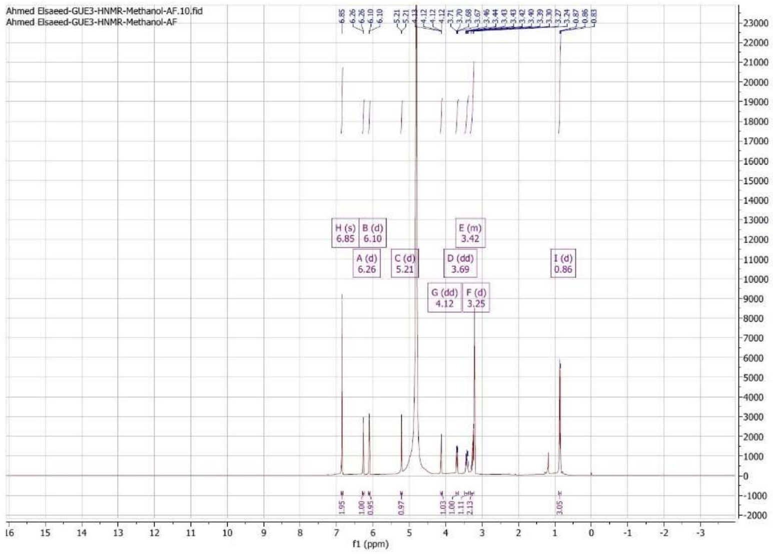

Figure S35: HNMR spectrum of compound 10 GUE3

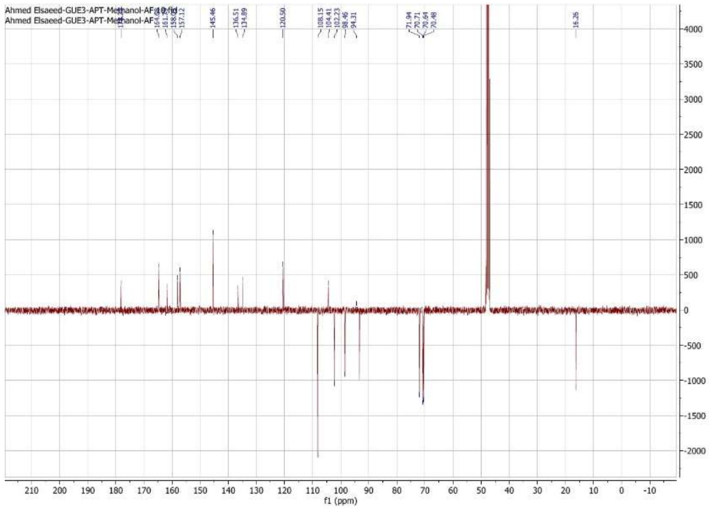

Figure S36: APT spectrum of compound 10 GUE3

Supplementary Materials

The effect of *G. abyssinica* extract and isolated compounds on accuracy of the glucose concentration assay.

Methods

Determination of the effect *G. abyssinica* extract and isolated compounds on accuracy of the glucose concentration assay

The Dinitrosalicylic acid reagent for Determination of glucose was used to assess the effect of tested compounds on the accuracy of glucose concentration assay of glucose. Briefly, 100 µL of DNS reagent was added to 150 µL of standard glucose (100 mM) and 150 µL of sample (2 mg/mL), was boiled at100 °C for 5 min, then cooled to room temperature. A sodium sulfate (100 µL; 0.5% w/v) was added to obtain stable color. Absorbance of reaction mixtures was measured spectrophotometrically at 540 nm (Spectro UV–VIS Auto, UV2602, Labomed, USA). Sample in mixture without test compounds or extracts were included as negative control [33].

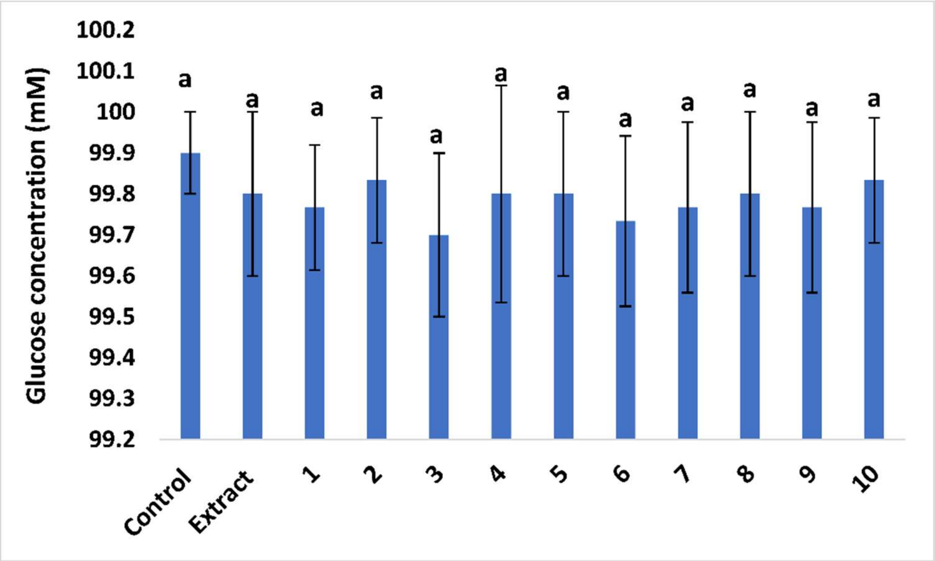

Figure S37. The effect of *G. abyssinica* extract and isolated compounds on accuracy of the glucose concentration assay. lower cases show statistical significance ($p < 0.05$)
